# Varicella zoster virus productively infects human peripheral blood mononuclear cells to modulate expression of immunoinhibitory proteins and blocking PD-L1 enhances virus-specific CD8^+^ T cell effector function

**DOI:** 10.1371/journal.ppat.1007650

**Published:** 2019-03-14

**Authors:** Dallas Jones, Christina N. Como, Lichen Jing, Anna Blackmon, Charles Preston Neff, Owen Krueger, Andrew N. Bubak, Brent E. Palmer, David M. Koelle, Maria A. Nagel

**Affiliations:** 1 Department of Neurology, University of Colorado School of Medicine, Aurora, Colorado, United States of America; 2 Department of Medicine, University of Washington, Seattle, Washington, United States of America; 3 Department of Medicine, Division of Allergy and Clinical Immunology, University of Colorado School of Medicine, Aurora, Colorado, United States of America; 4 Department of Laboratory Medicine, University of Washington, Seattle, Washington, United States of America; 5 Department of Global Health, University of Washington, Seattle, Washington, United States of America; 6 Vaccine and Infectious Disease Division, Fred Hutchinson Cancer Center, Seattle, Washington, United States of America; 7 Benaroya Research Institute, Seattle, Washington, United States of America; 8 Department of Ophthalmology, University of Colorado School of Medicine, Aurora, Colorado, United States of America; University of North Carolina at Chapel Hill, UNITED STATES

## Abstract

Varicella zoster virus (VZV) is a lymphotropic alpha-herpesvirinae subfamily member that produces varicella on primary infection and causes zoster, vascular disease and vision loss upon reactivation from latency. VZV-infected peripheral blood mononuclear cells (PBMCs) disseminate virus to distal organs to produce clinical disease. To assess immune evasion strategies elicited by VZV that may contribute to dissemination of infection, human PBMCs and VZV-specific CD8^+^ T cells (V-CD8^+^) were mock- or VZV-infected and analyzed for immunoinhibitory protein PD-1, PD-L1, PD-L2, CTLA-4, LAG-3 and TIM-3 expression using flow cytometry. All VZV-infected PBMCs (monocytes, NK, NKT, B cells, CD4^+^ and CD8^+^ T cells) and V-CD8^+^ showed significant elevations in PD-L1 expression compared to uninfected cells. VZV induced PD-L2 expression in B cells and V-CD8^+^. Only VZV-infected CD8^+^ T cells, NKT cells and V-CD8+ upregulated PD-1 expression, the immunoinhibitory receptor for PD-L1/PD-L2. VZV induced CTLA-4 expression only in V-CD8^+^ and no significant changes in LAG-3 or TIM-3 expression were observed in V-CD8^+^ or PBMC T cells. To test whether PD-L1, PD-L2 or CTLA-4 regulates V-CD8^+^ effector function, autologous PBMCs were VZV-infected and co-cultured with V-CD8^+^ cells in the presence of blocking antibodies against PD-L1, PD-L2 or CTLA-4; ELISAs revealed significant elevations in IFNγ only upon blocking of PD-L1. Together, these results identified additional immune cells that are permissive to VZV infection (monocytes, B cells and NKT cells); along with a novel mechanism for inhibiting CD8^+^ T cell effector function through induction of PD-L1 expression.

## Author summary

The burden of disease produced by VZV is significant, since 90% of the world population harbors latent virus. At least 50% of infected individuals will reactivate by 85 years of age to develop zoster, which is an established risk factor for stroke and myocardial infarction, as well as multisystem diseases with or without rash. VZV-infected PBMCs disseminate virus to distal organs to produce clinical disease. PD-L1 is an immunoinhibitory protein that interacts with PD-1, its receptor, expressed mainly on T cells to prevent their activation and subsequent clearance of virus- or malignantly transformed cells. We show here that all peripheral blood mononuclear cells have a dramatic induction of PD-L1 expression upon infection with VZV, which is combined with the induction of PD-1 expression on CD8^+^ T cells, NKT cells and VZV-specific CD8^+^ T cells. Blocking PD-L1 during co-culturing of VZV-specific CD8^+^ T cells with autologous VZV-infected PBMCs enhanced IFNγ levels almost 2-fold compared to isotype controls. These results indicate that blocking PD-L1 expression during varicella or zoster may restore CD8^+^ T cell effector function, enabling effective clearance of virus-infected cells to reduce viral spread and multisystem disease.

## Introduction

Varicella zoster virus (VZV) is a lymphotropic, human alpha-herpesvirinae subfamily member that produces varicella (chickenpox) upon primary infection, after which virus establishes latency in ganglionic neurons along the entire neuraxis [1]. With aging or immunosuppression, such as seen in patients with HIV, cancer or immunomodulatory therapies, VZV reactivates to produce herpes zoster (shingles), vascular disease and vision loss. The burden of disease produced by VZV is significant, since 90% of the world population harbors latent virus and at least 50% will experience reactivation by 85 years of age to develop zoster [2]. Zoster can be complicated by postherpetic neuralgia and is an established risk factor for stroke and myocardial infarction [3]. VZV infection of human peripheral blood mononuclear cells (PBMCs) is an essential step in virus dissemination and clinical disease. Following inhalation of viral particles during primary infection, infected PBMCs in the tonsils carry virus to skin to produce varicella rash [1]; virus can also spread to other organs to cause glomerulonephritis, abdominal pain and hepatitis [4, 5]. During reactivation, VZV spreads transaxonally and is also detected in PBMCs, albeit at a low rate with 1/10,000–100,000 PBMCs that are infected [6]; however, in immunosuppressed patients with disseminated zoster (cancer or HIV), rates of viremia may be higher [7].

The mechanism(s) that facilitates spread of VZV-infected PBMCs without effective immune clearance is unknown; however, upregulation of immunoinhibitory proteins may be important. Therefore, we hypothesized that VZV infection of PBMCs induces immunoinhibitory proteins to evade immune clearance. Such cell surface proteins normally act to halt an immune response after clearance of damaged or infected cells so that healthy cells are not harmed by inflammatory cytokines or activated immune cells [8–10]. For example, programmed cell death protein-1 (PD-1), a receptor expressed exclusively on immune cells and mainly T cells, is induced during T cell activation and chronic infections [9]. PD-1 elicits its immunoinhibitory function by binding with programmed death ligand-1 (PD-L1) or PD-L2, both of which can be expressed on virtually all cells, to inhibit positive signaling through the T cell receptor (TCR) and subsequent cytokine secretion [9, 10]. Cytotoxic T lymphocyte-associated protein-4 (CTLA-4) inhibits T cell function by binding to the T cell co-receptors CD80 and CD86 with a stronger affinity than that to the stimulatory co-receptor CD28 [11, 12]. Lymphocyte activation gene-3 (LAG-3) inhibits T cell activation since it binds with a higher affinity to MHC-II than CD4 [13] and also inhibits CD8^+^ T cell function during chronic viral infection [14]. Finally, T cell immunoglobulin and mucin-domain-containing-3 (TIM-3) inhibits TCR signaling and IL-2 secretion through interaction with its ligand galectin-9 [15].

Cancer- and virus-mediated upregulation of these immunoinhibitory proteins to evade clearance is well-established. Cancer cells induce the expression of immunoinhibitory ligands, such as PD-L1 and PD-L2, in the tumor microenvironment to prevent immune cell activation, enabling progression or metastasis. In that context, therapeutic targeting of immunoinhibitory proteins, mainly PD-1/PD-L1 and CTLA-4, has revolutionized cancer therapy [8]. Herpes simplex virus-1 (HSV-1), another alpha-herpesvirinae subfamily member, induces PD-1 expression during infection, and blockade of PD-L1 in mice enhanced primary and secondary CD8^+^ T cell immune responses [16]. PD-L1 expression was induced in HSV-1-infected neurons in mice and limited the survival of CD8^+^ T cells [17]. In addition, recombinant HSV-1 overexpressing CD80 permitted dendritic cell viral replication through binding of PD-L1 that resulted in enhanced T cell activation in mice [18]. While Epstein-Barr virus (EBV), a gammaherpesvirinae subfamily member, has a tropism for B cells and can transform them into Hodgkin’s lymphoma or Burkitt’s lymphoma cells, the combined blockade of PD-1 and CTLA-4 in humanized mouse models of EBV prevented lymphomagenesis [19]. Finally, HIV infection is associated with expression of several immunoinhibitory proteins on HIV-specific CD4^+^ and CD8^+^ T cells, such as PD-1, CTLA-4, TIM-3 and LAG-3 [20–23].

While earlier studies showed that VZV infection of human tonsillar T cells induces PD-1 expression [24] and that zoster patients have elevated PD-1 expression in T cells during reactivation [25], gaps remain in our knowledge of VZV dissemination via infected PBMCs. Thus, the permissiveness of PBMC subsets for VZV infection, the ability of virus to differentially modulate expression of immunoinhibitory proteins in a cell type-specific manner, the possible effects of viremia on uninfected bystander PBMCs and the clinical implications are still unclear. Understanding how VZV modulates immunoinhibitory proteins during viremia may have therapeutic value, which restores the ability of immune cells to clear virus infection and prevent hematogenous spread to other organs. Here, we analyzed both the ability of VZV to infect multiple immune cell subsets and the alterations in expression of immunoinhibitory proteins (PD-1/PD-L1/ PD-L2/CTLA-4/ LAG-3/TIM-3) in virus- and uninfected human PBMCs.

## Results

### VZV is able to infect human monocytes, NK cells, NKT cells, B cells, CD4^+^ T cells and CD8^+^ T cells in peripheral blood

**Fig 1:**
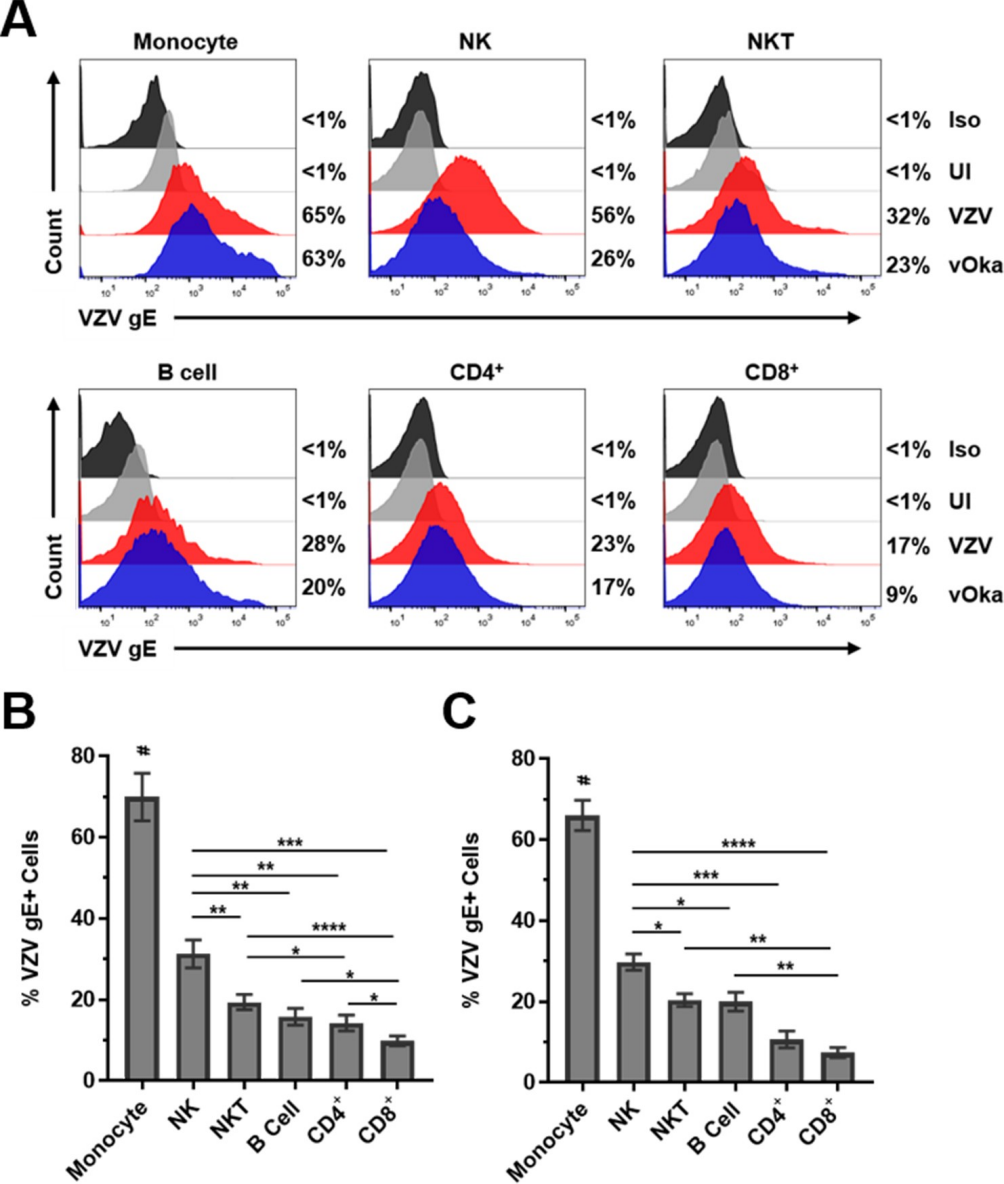
VZV infection of human monocytes, B cells, NK cells, NKT cells, CD4^+^ T cells and CD8^+^ T cells. **(A)** Human PBMCs were co-cultured with uninfected- (UI) or VZV-infected HFLs for 48 h then harvested and analyzed using flow cytometry. Specific cell types expressed the following markers: monocytes = CD3^-^CD56^-^CD19^-^CD14hiMHC-II^+^; B cells = CD3^-^CD56^-^CD19^+^; NK cells = CD3^-^CD56^+^; NKT cells = CD3^+^CD56^+^; CD4^+^ T cells = CD3^+^CD4^+^CD8^-^, and; CD8^+^ T cells = CD3^+^CD8^+^CD4^-^. Black histograms represent isotype control (Iso) stained VZV-infected cells, grey histograms represent UI cells, red histograms represent VZV-Ellen-infected cells (VZV) and blue histograms represent vOka-infected cells (vOka). Numbers on right side of each graph represent % VZV glycoprotein E-positive (gE+) cells. **(B-C)** Summary of flow cytometry analyses above from VZV-Ellen **(B)** and vOka **(C)** infections from 12 and 5 different healthy individuals, respectively, with bar graphs representing average % VZV-gE+ immune cells ± SEM. *P<0.05, **P<0.01, ***P<0.001, ****P<0.0001; # above monocytes represents P<0.01 **(B)** and P<0.0005 **(C)** for significant increases in % VZV-gE+ monocytes compared to all other immune cell populations analyzed. Statistical significance was determined using RM one-way ANOVA with the Greenhouse-Geisser correction and Tukey posttest.

To determine the permissiveness of immune cells to VZV infection, human PBMCs were infected with cell-associated VZV (Ellen strain) or vaccine strain (vOka) and analyzed 48 h later for expression of VZV surface glycoprotein E (gE) using flow cytometry (Fig 1A). The cell associated requirement for viral transmission of VZV is well known and this is an established method for infecting human immune cells with VZV [26, 27]. Flow cytometry gating scheme for individual immune cells is provided (S1 Fig). Results from PBMCs from 12 different healthy donors infected with VZV Ellen showed that monocytes were the most permissive to VZV infection (mean: 70%, range: 35–97%) followed by NK cells (mean: 32%, range: 12–61%), NKT cells (mean: 19%, range: 12–32%), B cells (mean: 16%, range: 7–29%), CD4^+^ T cells (mean: 14%, range: 5–29%) and CD8^+^ T cells were the least permissive to infection (mean: 10%, range: 4–17%) (Fig 1B and S1 Table). Monocytes showed a significantly higher infection rate when compared to all other immune cells analyzed (Fig 1B). Next, NK cells showed significantly higher levels of infection compared to all other immune cells analyzed with the exception of monocytes; NKT cells showed significantly higher levels of infection compared to both CD4^+^ and CD8^+^ T cells; B cells and CD4^+^ T cells showed significantly higher levels of infection compared to CD8^+^ T cells (Fig 1B). Results from PBMCs from 5 different healthy donors infected with vOka showed similar trends in permissiveness to VZV infection as the Ellen strain with monocytes having significantly higher infection rates when compared to all other immune cells analyzed, NK cells having significantly higher infection rates than all immune cells analyzed aside from monocytes, and NKT cells and B cells having significantly higher infection rates than CD8^+^ T cells (Fig 1C and S2 Table).

To confirm cell surface staining of VZV gE, we infected human PBMCs using cell-associated GFP-expressing strain of VZV (VZV-GFP). Aside from B cells being slightly more permissive to VZV-GFP infection than NKT, the preferential infection rates of VZV-GFP were similar to surface staining of VZV gE shown using both Ellen and vOka strains (S2 Fig and S3 Table), further supporting our observations.

To assess changes in VZV-infected PBMCs over time we co-cultured human PBMCs from 4 healthy individuals with uninfected- or VZV-infected HFLs (Ellen strain) and harvested PBMCs at 24, 48 and 72 h post infection (hpi) and analyzed for surface VZV-gE expression. PBMCs were co-cultured on separate 10cm^2^ petri dishes for each time point with VZV-infected HFLs that were infected with identical titers to allow the entire PBMC populations to be harvested and analyzed. Monocytes were 87%, 88% and 71% VZV-gE+ at 24, 48 and 72 hpi with a significant decrease in surface VZV-gE expression from 48 and 72 hpi (P = 0.006) (S3 Fig and S4 Table). B cells were 30%, 14% and 8% VZV-gE+ at 24, 48 and 72 hpi with significant decreases in surface VZV-gE expression at 24 hpi compared to both 48 and 72 hpi (P = 0.04 and 0.008, respectively) (S3 Fig and S4 Table). Interestingly, the remaining PBMC subsets showed slight increases in surface VZV-gE expression from 24 hpi to 48 hpi which was maintained at 72 hpi as well (S3 Fig and S4 Table); yet no statistically significant changes were observed. NK cells were 19%, 23% and 22% VZV-gE+ at 24, 48 and 72 hpi; NKT cells were 11%, 17% and 17% VZV-gE+ at 24, 48 and 72 hpi; CD4^+^ T cells were 9%, 17% and 17% VZV-gE+ at 24, 48 and 72 hpi; and CD8^+^ T cells were 6%, 10% and 11% VZV-gE+ at 24, 48 and 72 hpi.

To assess for viral expression levels of VZV in PBMC subsets we gated individual immune cells based upon VZV-gE expression levels. VZV-gE+lo cells were defined as having VZV-gE expression levels of log0-1 and VZV-gE+hi cells >log1 at 48 hpi. Monocytes had the highest % of VZV-gE+hi cells (44.5%) which was significantly higher than all other PBMC subsets analyzed aside from B cells (S4 Fig). B cells had 29.7% VZV-gE+hi cells which was significantly higher than NKT cells, CD8^+^ T cells and CD4^+^ T cells (S4 Fig). NK cells had 21.3% VZV-gE+hi cells, NKT cells had 16.3% VZV-gE+hi cells, CD8^+^ T cells had 10.8% VZV-gE+hi cells and CD4^+^ T cells had 8.3% VZV-gE+hi cells (S4 Fig).

Taken together, these results indicate that both the VZV Ellen and vaccine strain can infect all immune cell populations in the PBMC pool with preferential infection of monocytes, followed by NK cells, NKT cells and B cells; with CD4^+^ and CD8^+^ T cells being the least permissive to infection.

### VZV-infected monocytes, NK cells, NKT cells, B cells, CD4^+^ T cells, CD8^+^ T cells and HFLs all express nuclear VZV ORF63

**Fig 2:**
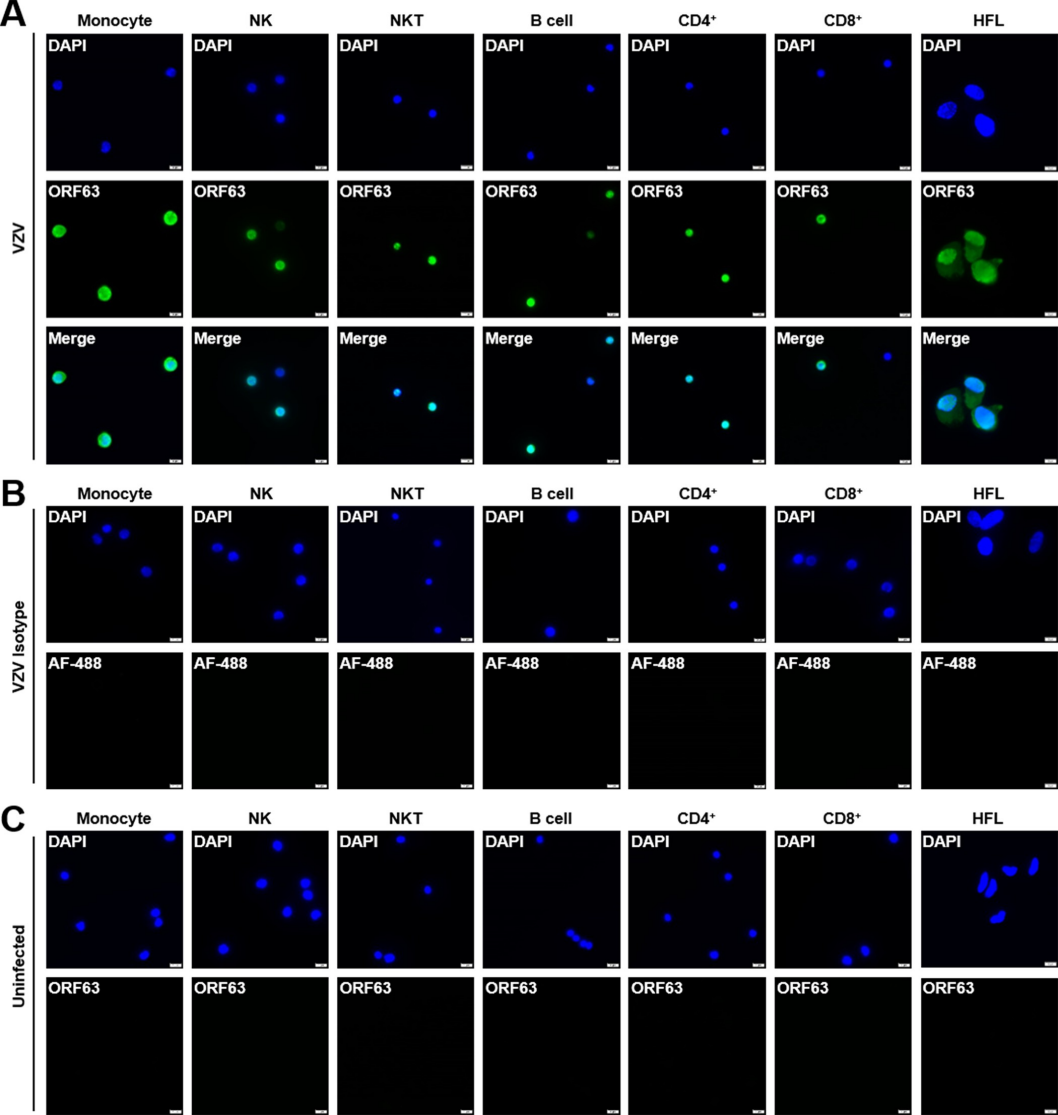
VZV-infected monocytes, NK cells, NKT cells, B cells, CD4^+^ T cells and CD8^+^ T cells all express nuclear VZV ORF63. Human PBMCs were co-cultured with uninfected- or VZV-infected HFLs for 48 h, then uninfected- and VZV-infected monocytes, NK, NKT, B cells, CD4^+^ T and CD8^+^ T cells based upon surface VZV gE expression were sorted using flow cytometry and analyzed for VZV ORF63 expression using immunofluorescence. Uninfected- and VZV-infected HFLs were analyzed for ORF63 expression as well. **(A)** VZV-infected monocytes, NK cells, NKT cells, B cells, CD4^+^ T cells, CD8^+^ T cells and HFLs all express nuclear VZV ORF63 (green). **(B)** VZV-infected monocytes, NK cells, NKT cells, B cells, CD4^+^ T cells, CD8^+^ T cells and HFLs were stained with Alexa Fluor-488 (AF-488, green) only as an isotype control for ORF63 expression. **(C)** Uninfected monocytes, NK cells, NKT cells, B cells, CD4^+^ T cells, CD8^+^ T cells and HFLs have no VZV ORF63 expression (green). DAPI (blue) was used as cell nucleus stain. Magnification, X400. Size bar = 10μm. Results representative of duplicate experiments using PBMCs from 2 different healthy donors.

To confirm cell surface flow cytometry VZV-gE expression in human PBMCs, PBMC subsets from 2 healthy donors were sorted using flow cytometry and stained for nuclear VZV ORF63 expression using immunofluorescence. As a positive control for ORF63 expression VZV-infected HFLs were analyzed as well. All VZV-infected monocytes, NK cells, NKT cells, B cells, CD4^+^ T cells, CD8^+^ T cells and HFLs showed nuclear VZV ORF63 expression indicative of productive infection (Fig 2A) that was not observed with isotype control stains (Fig 2B), while uninfected PBMCs and HFLs showed no ORF63 expression as expected (Fig 2C). Taken together, these results confirm that all VZV-infected PBMC subsets express nuclear VZV ORF63.

### VZV-infected monocytes, NK cells, NKT cells, B cells, CD4^+^ T cells and CD8^+^ T cells express immediate early (ORF63) and late (ORF68) VZV transcripts

**Fig 3:**
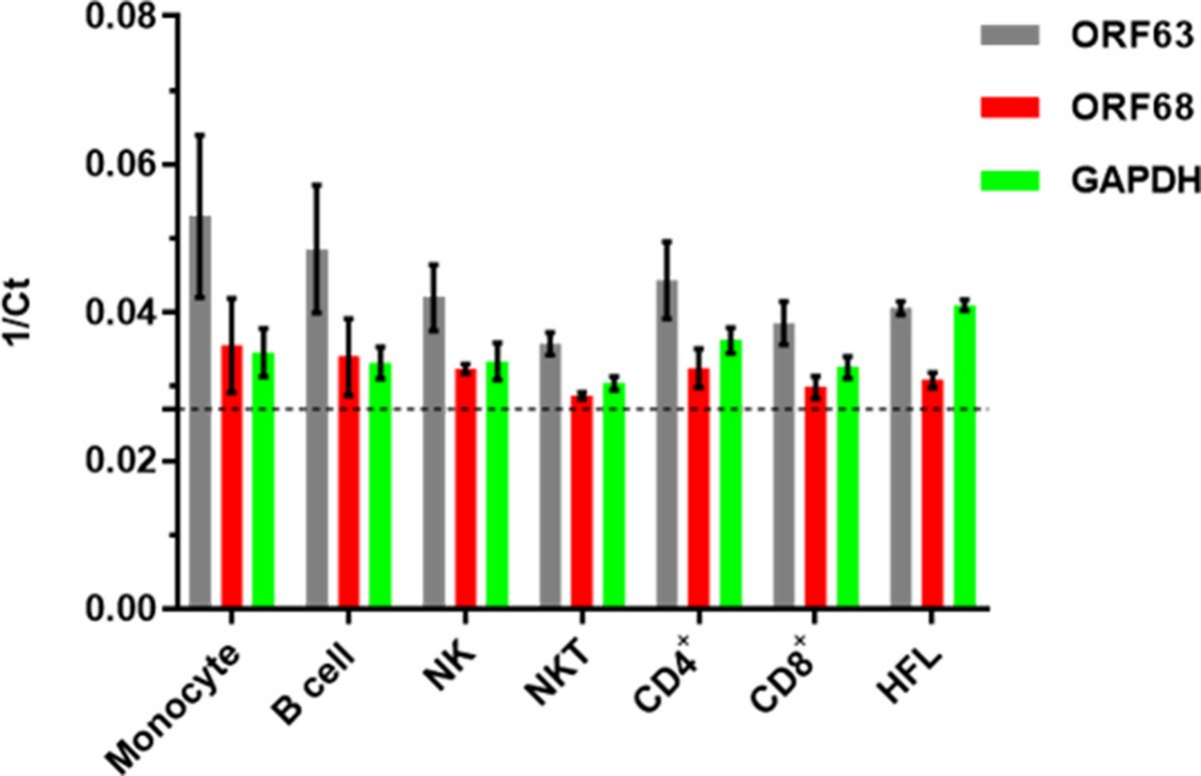
Quantitative RT-PCR analyses of GAPdH, VZV ORF63 and ORF68 expression in VZV-infected monocytes, NK cells, NKT cells, B cells, CD4^+^ T cells, CD8^+^ T cells and HFLs. Human PBMCs were infected with VZV as described in Fig 1, then uninfected- and VZV-infected monocytes, NK cells, NKT cells, B cells, CD4^+^ T cells and CD8^+^ T cells were sorted using flow cytometry. RNA was harvested and then analyzed by quantitative RT-PCR for *ORF63* (grey bars), *ORF68* (red bars) and GAPdH (green bars) transcripts. In addition, VZV-infected HFLs that were >90% VZV gE+ based upon flow cytometry analyses were harvested as a positive control for viral transcript expression. Results representative of sorted PBMCs from 3 different healthy individuals and from 3 independent HFL infections, with bar graphs representing average 1/Ct ± SD. Dotted line represents threshold for Ct values (1/36). All immune cell populations were >93% pure based upon flow cytometry sorting.

The productive replication cycle of all herpesviruses occurs through an orderly chronological cascade of gene expression composed of immediate early, early and late genes [28]. To confirm that the flow cytometry findings showing VZV gE expression on infected PBMCs represented productive virus infection, viral transcripts were examined in each of these VZV-infected cell populations. Specifically, PBMCs were infected with VZV for 48 h, immune cells were sorted and determined to be >93% pure based upon flow cytometry analyses. RNA was extracted and q-RT-PCR analyses for immediate early (ORF63) and late (ORF68) viral transcripts along with the housekeeping gene GAPdH from uninfected and VZV-infected PBMCs were performed (n = 3 healthy donors). A threshold of 36 for Ct values for all transcripts was set and data analyzed using the 1/Ct method (threshold = 0.028) (Fig 3). All VZV-infected immune cell populations analyzed showed expression of both immediate early and late viral transcripts along with GAPdH (Fig 3). As a control for productive viral transcript expression, VZV-infected HFLs that were >90% VZV gE+ based upon flow cytometry analyses were harvested and subjected to the same q-RT-PCR analyses as PBMCs. VZV-infected HFLs had similar expression levels of ORF63 and ORF68 transcripts when compared to all PBMC subsets (Fig 3), further confirming that PBMCs are productively infected by VZV. In addition, VZV-infected immune cell populations and HFLs were analyzed for q-RT-PCR analyses without reverse transcriptase following DNase treatment and Ct values were undetermined for both ORF63 and GAPdH (S5 Table), indicating no contamination from viral DNA. Uninfected immune cells were also sorted and analyzed for viral transcripts and GAPdH, no expression of viral transcripts was observed (S5 Table). Taken together, these results support that VZV can productively infect monocytes, NK cells, NKT cells, B cells, CD4^+^ T cells and CD8^+^ T cells.

### Monocytes, NK cells, NKT cells, B cells, CD4^+^ T cells and CD8^+^ T cells are productively infected by VZV and capable of transmitting virus

**Fig 4:**
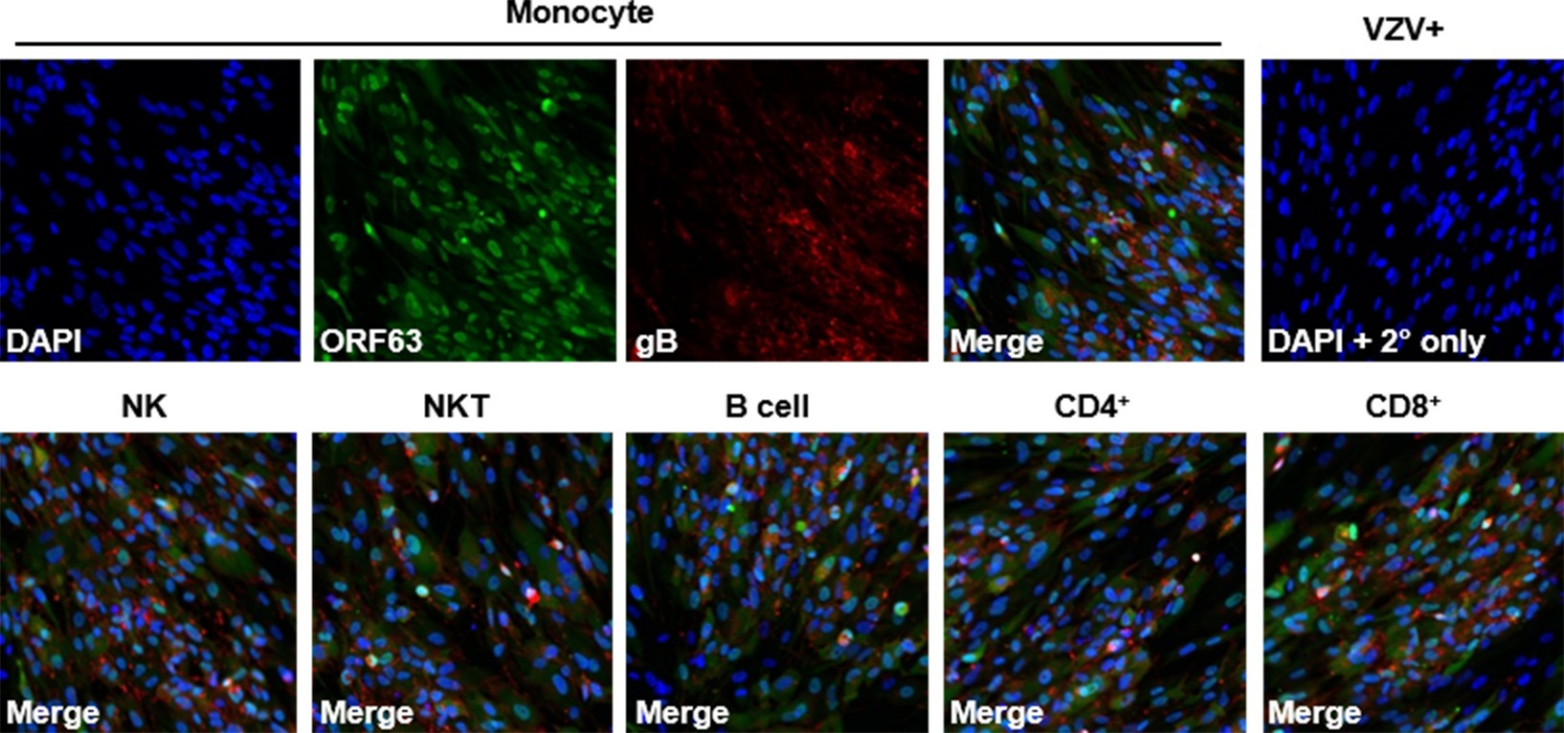
Monocytes, NK cells, NKT cells, B cells, CD4^+^ T cells and CD8^+^ T cells are productively infected by VZV and capable of transmitting virus. Human PBMCs were co-cultured with VZV-infected HFLs for 48 h, then VZV-infected monocytes, NK, NKT, B cells, CD4^+^ T and CD8^+^ T cells were sorted using flow cytometry. Individual sorted immune cells were then washed with citrate buffer followed by FACS buffer before co-culturing with uninfected HFLs. After 5 days of co-culture, immunofluorescence analyses of VZV ORF63 (green) and VZV glycoprotein B (gB, red) revealed productive infection of HFLs by all individual immune cell populations analyzed. Specifically, top panels 1 to 3 (left to right) show individual channels for DAPI, ORF 63 and VZV gB in HFLs exposed to VZV-infected monocytes—with panel 4 representing a merged image demonstrating expression of both VZV proteins. Lower merged panels show co-expression of ORF 63 and VZV gB in HFLs exposed to VZV-infected NK cells, NKT cells, B cells, CD4^+^ T cells and CD8^+^ T cells. Negative controls were provided by omission of primary antibody on VZV-infected HFLs (top right panel). DAPI (blue) was used as cell nucleus stain. Magnification, X100. Results representative of duplicate experiments using PBMCs from 2 different healthy donors.

Since VZV infection causes a viremia upon primary infection and VZV-infected immune cells are how virus is disseminated to skin and other organs [1], we assessed if all VZV-infected immune cell subsets are capable of transmitting virus to another cell type. VZV-infected monocytes, NK cells, NKT cells, B cells, CD4^+^ T cells and CD8^+^ T cells from 2 different healthy donors were sorted using flow cytometry then washed with citrate buffer to remove any potential virus adhering to the cell surface that would not be transferred by a productively-infected immune cell as this is a commonly used method for ensuring productive infection by alphaherpes viruses [29]. Infected cells were then washed with FACS buffer and co-cultured with HFLs for 3 days. After 5 days, adherent HFLs were fixed and analyzed for ORF63 and VZV gB expression using immunofluorescence. All VZV-infected monocytes, NK cells, NKT cells, B cells, CD4^+^ T cells and CD8^+^ T cells were capable of transmitting virus to HFLs as demonstrated by the expression of both VZV ORF63 and VZV gB (Fig 4). Additionally, VZV infected monocytes, NK cells, NKT cells, B cells, CD4^+^ T cells and CD8^+^ T cells from 2 different healthy donors were sorted and co-cultured with HFLs without washing with citrate buffer and analyzed for VZV gE expression using flow cytometry after 5 days. All immune cells were capable of transmitting virus to HFLs as well (S5 Fig).

In summary, VZV infected monocytes, NK cells, NKT cells, B cells, CD4^+^ T cells and CD8^+^ T cells were all capable of transmitting virus to another cell type which supports the notion that during viremia, any of these cells are capable of carrying virus to the skin and distal organs.

### VZV-mediated regulation of PD-L1, PD-L2 and PD-1 expression in human monocytes, NK cells, NKT cells, B cells, CD4^+^ T cells and CD8^+^ T cells

**Fig 5:**
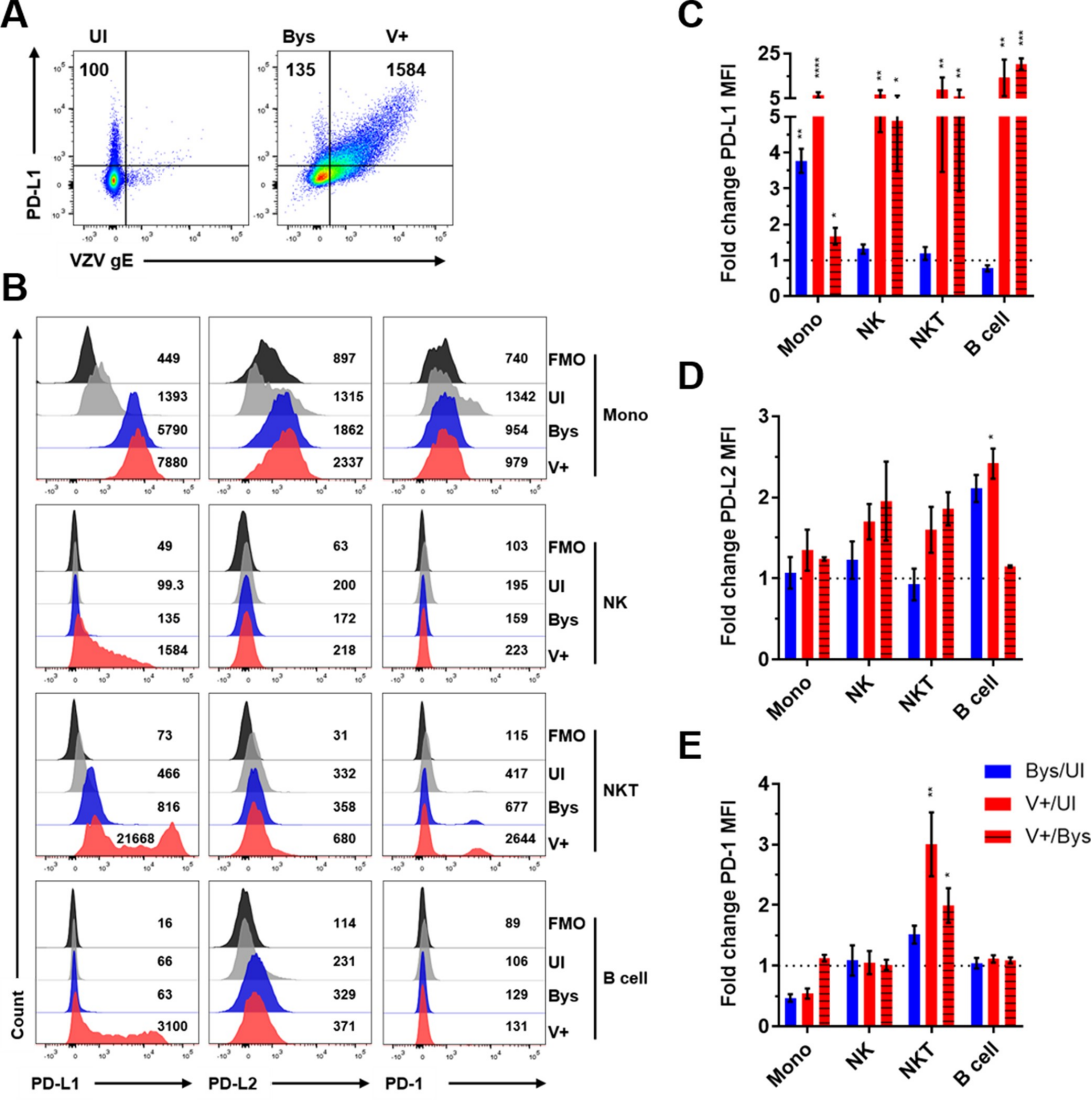
VZV-mediated regulation of PD-L1, PD-L2 and PD-1 expression in human monocytes, B cells, NK cells and NKT cells. Human PBMCs were co-cultured with uninfected- or VZV-infected HFLs for 48 h as described in Fig 1 then harvested and analyzed for PD-1, PD-L1, PD-L2 and VZV gE expression using flow cytometry. **(A)** Flow cytometry gating strategy for uninfected (UI), VZV gE-negative bystander (Bys) and VZV gE+ (V+) immune cell populations. **(B)** Representative flow cytometry plots of PD-L1, PD-L2 and PD-1 MFI expression levels in monocytes (Mono), NK cells, NKT cells and B cells. Black histograms = FMO control, grey histograms = UI, blue histograms = Bys and red histograms = V+ cells. **(C-E)** Summary of fold change in MFI in PD-L1 **(C)**, PD-L2 **(D)** and PD-1 **(E)** expression levels in: Bys/UI (blue), V+/UI (red) and V+/Bys (red with black stripes) with respect to all immune cell populations analyzed. Results are representative of eight independent experiments with PBMCs from eight healthy individuals. Bar graphs represent average fold-change in MFI ± SEM. *P<0.05, **P<0.01, ***P<0.001, ****P<0.0001. Statistical significance was determined using RM one-way ANOVA with the Greenhouse-Geisser correction and Tukey posttest.

**Fig 6:**
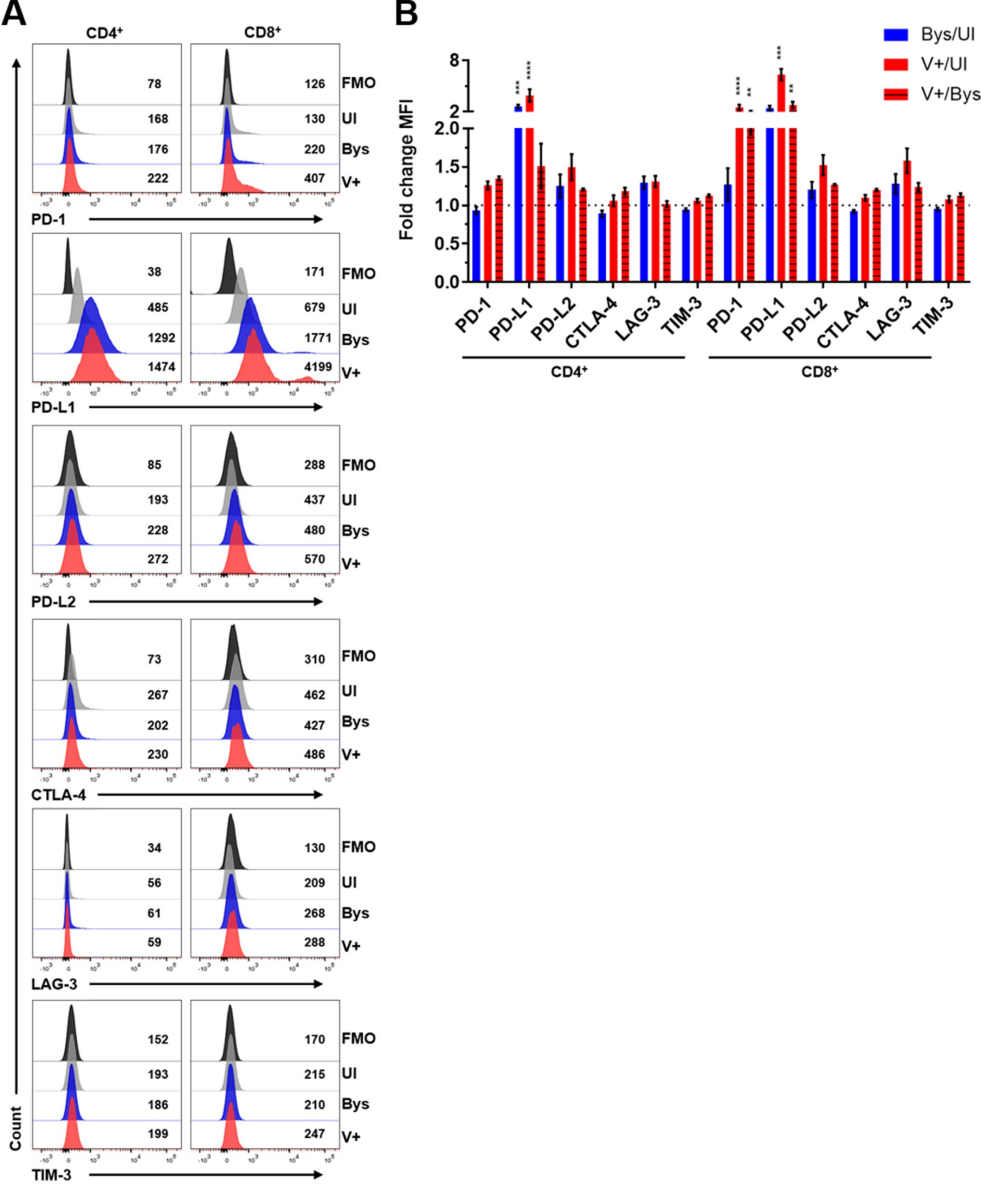
VZV-mediated regulation of PD-1, PD-L1, PD-L2, CTLA-4, LAG-3 and TIM-3 expression in human CD4^+^ and CD8^+^ T cells. Human PBMCs from Fig 4 were co-cultured with uninfected- or VZV-infected HFLs for 48 h as described in Fig 1 then harvested and analyzed for PD-1, PD-L1, PD-L2, CTLA-4, LAG-3, TIM-3 and VZV gE expression using flow cytometry. **(A)** Representative flow cytometry plots of PD-1, PD-L1, PD-L2, CTLA-4, LAG-3 and TIM-3 MFI expression levels in CD4^+^ and CD8^+^ T cells. Black histograms = FMO control, grey histograms = UI, blue histograms = Bys and red histograms = V+ cells. **(B)** Summary of fold change in MFI in PD-1, PD-L1, PD-L2, CTLA-4, LAG-3 and TIM-3 expression levels in: Bys/UI (blue), V+/UI (red) and V+/Bys (red with black stripes) with respect to CD4^+^ and CD8^+^ T cells. Results are representative of eight independent experiments with PBMCs from eight healthy individuals that are from the same experiments shown in Fig 4. Bar graphs represent average fold-change in MFI ± SEM. **P<0.01, ***P<0.001, ****P<0.0001. Statistical significance was determined using RM one-way ANOVA with the Greenhouse-Geisser correction and Tukey posttest.

The involvement of the PD-1:PD-L1 pathway in immune evasion during viral infection and carcinogenesis is well-established [9, 10]. PBMCs were co-cultured with VZV-infected HFLs and utilizing flow cytometry gating on VZV gE expression, the mean fluorescent intensity (MFI) levels of PD-L1, PD-L2 and PD-1 in uninfected immune cells (UI), VZV gE+ immune cells (V+) and VZV gE-negative bystander cells (Bys) was determined (Fig 5A). Immune cells were gated for immunoinhibitory protein expression using FMO controls (Figs 5B and 6A). All V+ immune cells had significant elevations in PD-L1 MFI levels when compared to their UI counterparts (Figs 5C and 6B). Aside from CD4^+^ T cells, all V+ immune cell populations analyzed had significant elevations in PD-L1 MFI levels compared to their Bys counterparts (Figs 5C and 6B). Only Bys monocytes and CD4^+^ T cells had significant elevations in PD-L1 MFI levels compared to UI counterparts (Figs 5C and 6B). PD-L2 MFI levels were significantly elevated in V+ B cells compared to UI cells (Fig 5D). Only V+ NKT cells and CD8^+^ T cells had statistically significant elevations in PD-1 MFI levels compared to both their UI and Bys counterparts (Figs 5E and 6B). To test for additional immunoinhibitory proteins that can be induced in CD4^+^ and CD8^+^ T cells, we examined CTLA-4, LAG-3 and TIM-3 expression since these pathways, like that of PD-1, can also suppress T cell activation [11, 14, 15]. No significant changes in CTLA-4, LAG-3 or TIM-3 expression levels were detected in either CD4^+^ or CD8^+^ T cells (Fig 6B). Statistical analyses of all immunoinhibitory proteins analyzed is provided (S4 and S6 Tables). As a control for immunoinhibitory protein staining, PBMCs were cultured alone and treated with or without PMA/Ionomycin and significant elevations of all immunoinhibitory proteins was observed in PMA/Ionomycin treated CD8^+^ T cells (S6 Fig).

In summary, all VZV-infected immune cells had significant elevations in PD-L1 expression when compared to their uninfected counterparts, while only VZV-infected NKT cells and CD8^+^ T cells had significant inductions in PD-1 expression when compared to both UI and Bys cells. PD-L2 was only significantly elevated in VZV-infected B cells compared to UI cells; indicating that the main immunoinhibitory pathway induced during VZV infection of PBMCs is the PD-1:PD-L1 pathway.

### VZV ORF34- and ORF18-specific CD8^+^ T cells recognize VZV-infected HBVAFs/HFLs and are prone to infection

**Fig 7:**
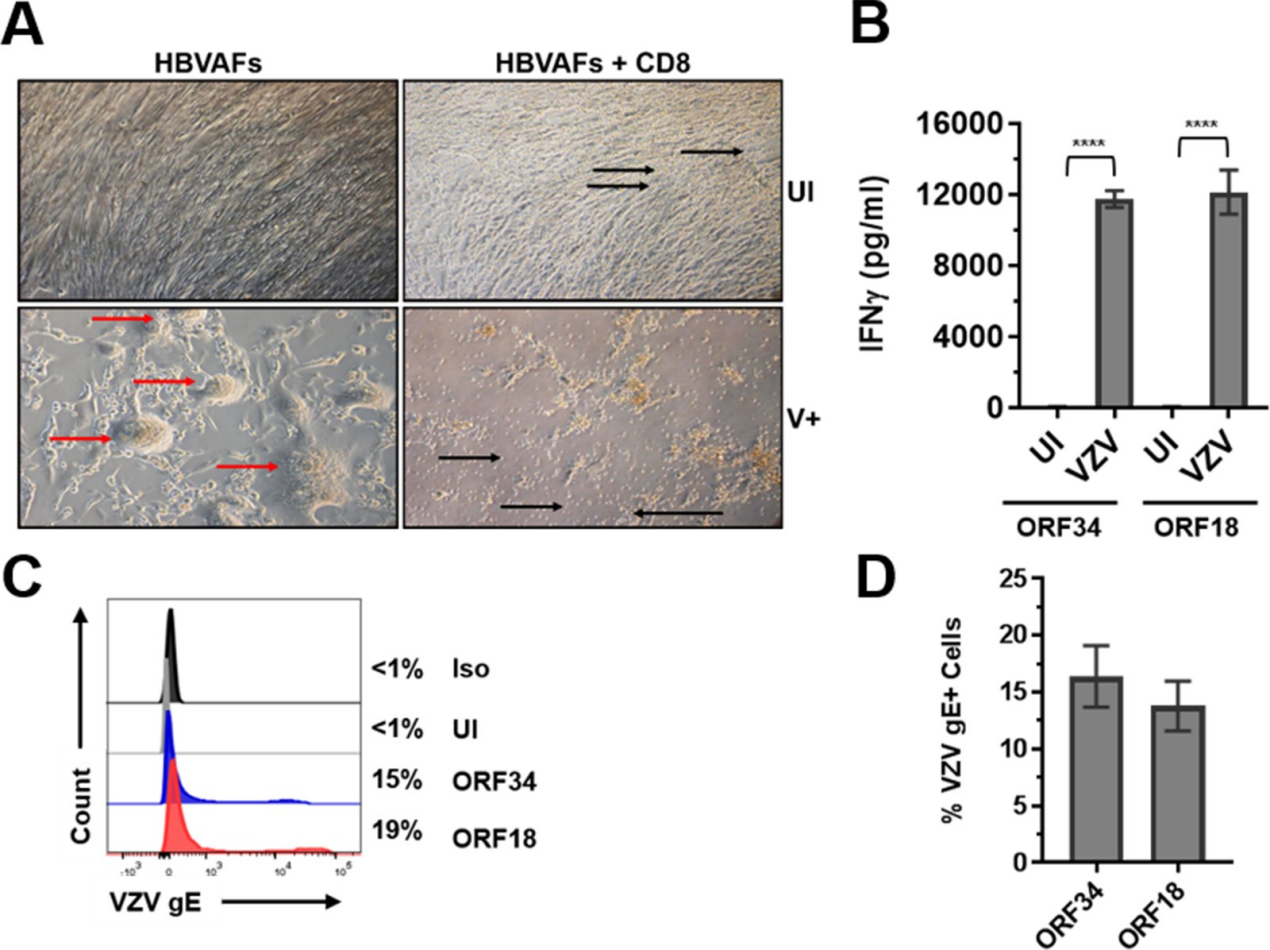
VZV ORF34- and ORF18-specific CD8^+^ T cells recognize VZV-infected HBVAFs and are prone to infection. **(A)** HBVAFs were uninfected- (UI) or VZV-infected (V+) for 72 h and then cultured alone or with VZV ORF34- or ORF18-specific CD8^+^ T cells for an additional 48 h. After 48 h of co-culturing, phase contrast microscopy images were taken. Black arrows represent CD8^+^ T cells and red arrows represent VZV-infected region of HBVAFs. Magnification, X100. Representative images of 4 independent experiments. **(B)** Cell culture supernatants were harvested 24 h after co-culturing VZV ORF34- or ORF18-specific CD8^+^ T cells with UI- or VZV-infected HBVAFs/HFLs and analyzed for IFNγ levels using ELISA. Results representative of 8 independent experiments with bar graphs representing average IFNγ levels (pg/ml) ± SEM. Statistical significance was determined using the unpaired Student’s *t*-test. ****P<0.0001. **(C)** Representative flow cytometry analyses of VZV gE expression in live CD8^+^ T cells 48 h after co-culturing. Black histograms = Isotype control (Iso), grey = UI cells, blue histograms = VZV-infected ORF34 cells, red histograms = VZV-infected ORF18 cells. Numbers represent % of cells VZV gE+. **(D)** Summary of flow cytometry analyses of % VZV gE+ cells in live CD8^+^ ORF34- or ORF-18 specific T cells. Results representative of 19 and 16 independent experiments from VZV ORF18- and ORF34-specific CD8^+^ T cells, respectively. Bar graphs represent average % of cells VZV gE+ ± SEM.

VZV-specific CD8^+^ T cells that recognize discrete HLA A*0201-restricted nonamer epitopes in either VZV ORF34 or ORF18 proteins have been enriched and characterized [30]. These cells are HLA compatible with both HFLs and HBVAFs used in our experiments as confirmed by staining with an HLA-A*0201-reactive monoclonal antibody. Uninfected- or VZV-infected HBVAFs were cultured for 72 h after infection; cells were then co-cultured without or with VZV ORF34- or ORF18-specific CD8^+^ T cells for 48 h and analyzed. Phase microscopy showed that in the absence of T cells, uninfected HBVAFs formed a confluent monolayer (Fig 7A, top left panel), whereas VZV-infected HBVAFs formed clusters (syncytia) of infected cells (Fig 7A, bottom left panel). In the presence of VZV-specific CD8^+^ T cells directed against ORF34 or ORF18, uninfected HBVAFs maintained a confluent monolayer (Fig 7A, top right panel), whereas VZV-infected HBVAFs had significant cell lysis and disruption of the clustered cells (Fig 7A, bottom right panel).

Concurrently, 24 h after exposure of uninfected- and VZV-infected HFLs/HBVAFs to VZV-specific CD8^+^ T cells, conditioned supernatant was collected and analyzed for IFNγ levels using ELISA. Both VZV ORF34- and ORF-18-specific CD8^+^ T cells had dramatic elevations in IFNγ levels when co-cultured with VZV-infected cells when compared to uninfected cells (Fig 7B, P<0.0001 for both), which further supports their ability to mount a cellular-mediated immune response towards VZV infection. To confirm that IFNγ secretions elicited by VZV ORF34- and ORF-18-specific CD8^+^ T cells were due to proper MHC-I recognition of VZV antigen we co-cultured uninfected- and VZV-infected HBVAFs with HLA-A*0201-restricted HSV-2 UL47 specific CD8^+^ T cells or HLA-B*0702-restricted HSV-2 UL49 specific CD8^+^ T cells during the same experiments. Virtually no IFNγ secretions were elicited by either CD8^+^ T cells specific for HSV-2 during co-culture with either uninfected- or VZV-infected HFLs (S7 Fig). Additionally, we confirmed our previous reports on VZV-mediated down-regulation of MHC-I in HBVAFs [31] and show that VZV-infected HBVAFs maintain significant expression levels of MHC-I when compared to isotype control stains (S8 Fig). Overall, our data indicate that VZV-infected HBVAFs are still capable of presenting antigen to VZV-specific CD8^+^ T cells. VZV-infected HBVAFs and uninfected HBVAFs had no autonomous IFNγ secretions when cultured alone which confirms the dramatic induction of IFNγ elicited by VZV-specific CD8^+^ T cells during co-culturing with VZV-infected HBVAFs (S7 Fig).

While the VZV ORF34- and ORF18-specific CD8^+^ T cells elicited a robust anti-viral immune response towards VZV-infected HBVAFs they were also permissive to VZV infection based upon flow cytometry analyses of VZV gE expression (Fig 7C). On average 16% of VZV-ORF34- and 14% of ORF18-specific CD8^+^ T cells expressed VZV gE after 48 h of co-culturing (Fig 7D).

Taken together, the VZV ORF34- and ORF18-specific CD8^+^ T cells induced robust levels of IFNγ secretions and recognition of VZV-infected HBVAFs/HFLs; importantly, these T cells were also prone to VZV infection based upon surface VZV gE expression.

### VZV-mediated induction of PD-1, PD-L1, PD-L2 and CTLA-4 expression in VZV ORF34- or ORF18-specific CD8^+^ T cells

**Fig 8:**
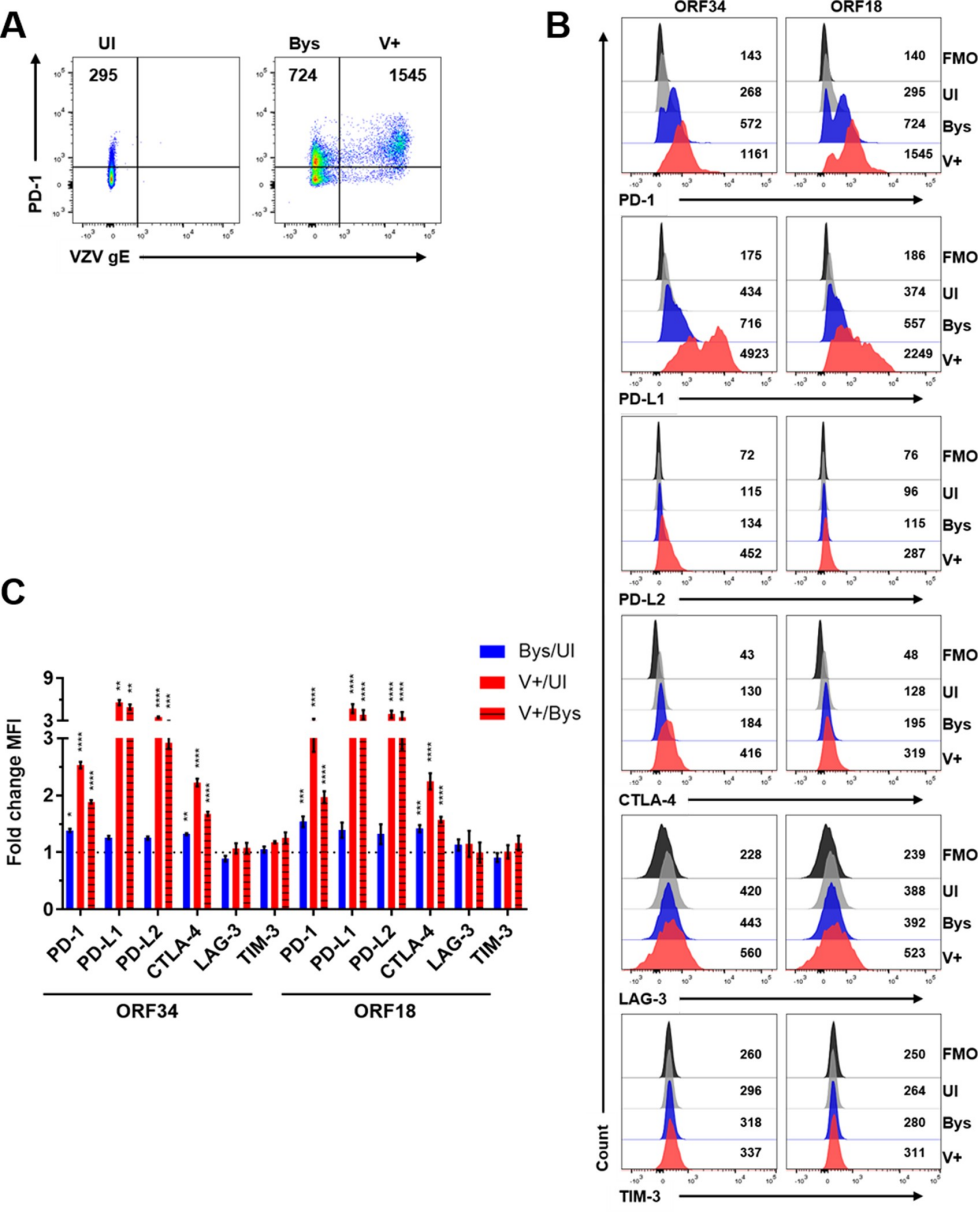
VZV-mediated induction of PD-1, PD-L1, PD-L2 and CTLA-4 expression in VZV ORF34- or ORF18-specific CD8^+^ T cells. VZV ORF34- or ORF18-specific CD8^+^ T cells were co-cultured with uninfected- or VZV-infected HFLs/HBVAFs for 48 h, harvested and analyzed for VZV-gE, PD-1, PD-L1, PD-L2, CTLA-4, LAG-3 and TIM-3 expression. Live CD8^+^ cells are shown. **(A)** Flow cytometry gating strategy for uninfected (UI), VZV gE-negative bystander (Bys) and VZV gE+ (V+) CD8+ T cells. **(B)** Representative flow cytometry plots of PD-1, PD-L1, PD-L2, CTLA-4, LAG-3 and TIM-3 MFI expression levels. Black histograms = FMO control, grey histograms = UI, blue histograms = Bys and red histograms = V+ cells. **(C)** Summary of fold change in MFI in PD-1, PD-L1, PD-L2, CTLA-4, LAG-3 and TIM-3 expression levels in: Bys/UI (blue), V+/UI (red) and V+/Bys (red with black stripes) with respect to ORF34- or ORF18-specific CD8^+^ T cells. Results are representative of 19, 16 and 12 independent experiments for PD-1/PD-L1, PD-L2/CTLA-4 and LAG-3/TIM-3 expression in VZV ORF18-specific CD8^+^ T cells, respectively, and of 14, 10 and 6 independent experiments for PD-1/PD-L1, PD-L2/CTLA-4 and LAG-3/TIM-3 expression in VZV ORF34-specific CD8^+^ T cells, respectively. Bar graphs represent average fold-change in MFI ± SEM. *P<0.05, **P<0.01, ***P<0.001, ****P<0.0001. Statistical significance was determined using RM one-way ANOVA with the Greenhouse-Geisser correction and Tukey posttest.

To assess for immunoinhibitory proteins induced in VZV ORF34- or ORF18-specific CD8^+^ T cells during clearance of VZV-infected HBVAFs/HFLs, we harvested cells as mentioned above and analyzed for PD-1, PD-L1, PD-L2, CTLA-4, LAG-3 and TIM-3 expression levels using flow cytometry. The same gating scheme of UI, Bys and V+ cells was used for analyses (Fig 8A). Cells were gated using an FMO control for all immunoinhibitory proteins analyzed and MFI levels were compared amongst the 3 populations (Fig 8B). All V+ cells in both VZV ORF34- and ORF18-specific CD8^+^ T cells had statistically significant elevations in PD-1, PD-L1, PD-L2 and CTLA-4 MFI levels compared to UI cells and Bys cells (Fig 8C). Additionally, all Bys cells in in both VZV ORF34- and ORF18-specific CD8^+^ T cells had statistically significant elevations in PD-1 and CTLA-4 MFI levels compared to UI cells (Fig 8C). No statistically significant elevations in LAG-3 or TIM-3 MFI levels were observed in either VZV ORF34- or ORF18-specific CD8^+^ T cells when compared amongst UI, Bys or V+ cells (Fig 8C). Statistical analyses of all immunoinhibitory proteins analyzed is provided (S7 Table). In addition, both VZV ORF34- and ORF18-specific CD8^+^ T cells had significantly higher percentages of VZV-gE+hi cells when compared to VZV-infected CD8^+^ T cells from healthy donor PBMCs (S4 Fig), which could explain the VZV-mediated induction of CTLA-4 and PD-L2 observed in these cells that was not observed in CD8^+^ T cells from healthy donor PBMCs.

In summary, both VZV-specific CD8^+^ T cell clones had significant inductions of PD-1, PD-L1, PD-L2 and CTLA-4 expression in VZV-infected cells when compared to uninfected cells; while a bystander effect for PD-1 and CTLA-4 induction was also observed in both clones.

### Only PD-L1 blockade enhances IFNγ secretion by VZV ORF34- or ORF18-specific CD8^+^ T cells during co-culture with autologous VZV-infected PBMCs

**Fig 9:**
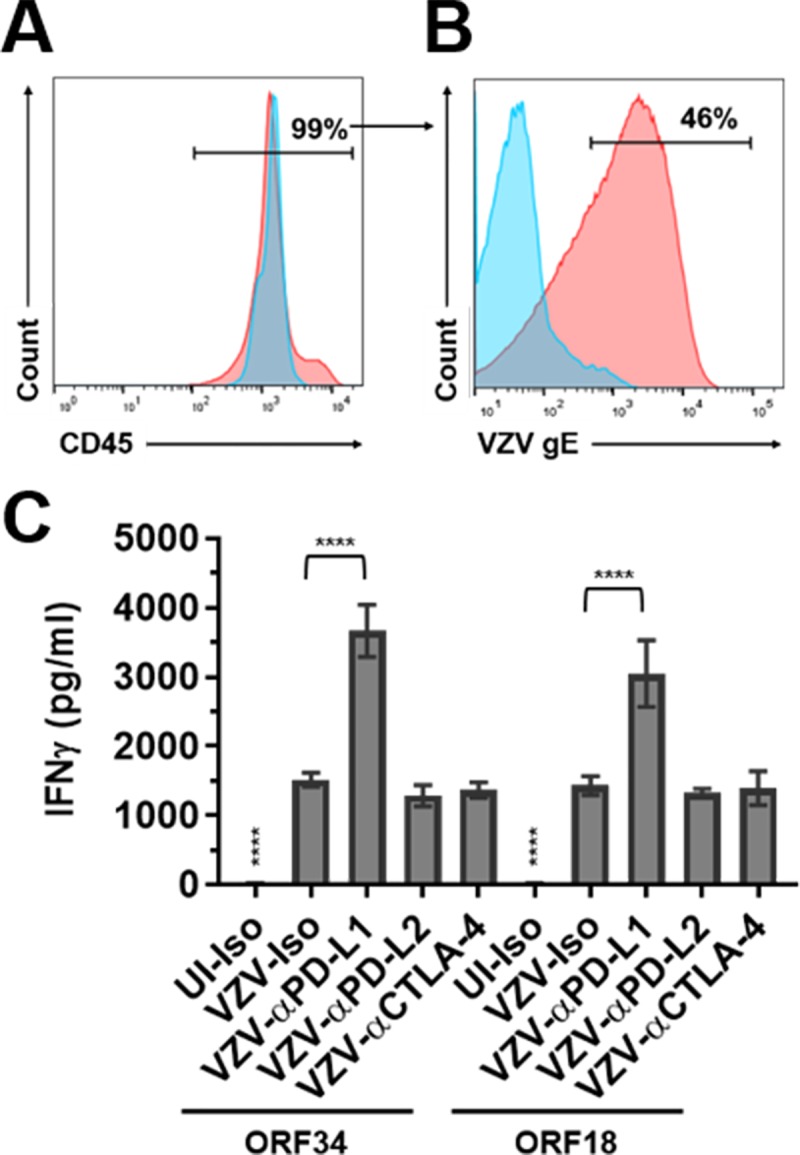
Only PD-L1 blockade enhances IFNγ secretion by VZV ORF34- or ORF18-specific CD8^+^ T cells during co-culture with autologous VZV-infected PBMCs. PBMCs from the same donor as the VZV ORF34- and ORF18-specific CD8^+^ T cells were co-cultured with uninfected- or VZV-infected HFLs for 48 h and then cells were harvested and analyzed by flow cytometry for percent CD45 purity **(A)** and percent VZV-gE positive **(B)** before co-culturing with VZV ORF34- or ORF18-specific CD8^+^ T cells. Blue histograms represent uninfected PBMCs and red histograms represent VZV-infected PBMCs. **(C)** Uninfected- (UI) or VZV-infected autologous PBMCs were co-cultured with VZV ORF34- or ORF18-specific CD8^+^ T cells in the presence of PD-L1 (αPD-L1), PD-L2 (αPD-L2) or CTLA-4 (αCTLA-4) blocking antibodies along with isotype controls (Iso). After 24 h, cell culture supernatants were harvested and analyzed for IFNγ levels using ELISA. Results from all experiments shown are representative of triplicate experiments, with bar graphs representing mean IFNγ levels (pg/ml) ± SD. **** above UI-Iso denotes P<0.0001 for increases in IFNγ levels in all VZV-infected co-cultures compared to uninfected co-cultures. **** above VZV-αPD-L1 denotes P<0.0001 for increase in IFNγ levels compared to VZV-infected isotype controls. Statistical significance was determined using RM one-way ANOVA with the Greenhouse-Geisser correction and Tukey posttest.

Since primary infection with VZV induces viremia, we infected cryopreserved/thawed PBMCs with VZV and co-cultured them with VZV ORF34- or ORF18-specific CD8^+^ T cells to mimic leukocyte-leukocyte interactions during the viremic phase of primary VZV infection. The ORF34- and ORF18-specific CD8^+^ effector T cells and VZV-infected PBMCs were all from the same donor. PBMCs were co-cultured with uninfected- or VZV-infected HFLs for 48 h, and non-adherent cells were harvested and analyzed for VZV infection of CD45+ cells using flow cytometry before co-culturing with VZV-specific CD8^+^ T cells. Both uninfected- and VZV-infected non-adherent cells were 99% CD45+ (Fig 9A), and VZV-infected PBMCs were approximately 45% VZV-gE+ (Fig 9B) based on flow cytometry analyses. VZV-infected autologous PBMCs were cultured with VZV ORF34- or ORF18-specific CD8^+^ T cells at a 1:1 ratio in the presence of anti-PD-L1 (αPD-L1), anti-PD-L2 (αPD-L2) or anti-CTLA-4 (αCTLA-4) blocking antibodies or isotype controls. Since no induction of LAG-3 or TIM-3 expression was observed these immunoinhibitory proteins were not blocked. After 24 h of co-culturing autologous PBMCs with VZV ORF34- or ORF18-specific CD8^+^ T cells, cell culture supernatants were harvested and analyzed for IFNγ levels using ELISA (Fig 9C). All VZV-infected PBMCs co-cultured with VZV ORF34- or ORF18-specific CD8^+^ T cells had significant elevations in IFNγ levels as compared to their uninfected counterparts (P<0.0001). Importantly, only blocking of PD-L1 induced significant elevations in IFNγ levels when compared to isotype controls (P<0.0001 for both). Virtually no changes in IFNγ levels were observed when PD-L2 or CTLA-4 was blocked compared to isotype controls for either VZV ORF34- or ORF18-specific CD8^+^ T cells co-cultured with VZV-infected PBMCs. Also, cell culture supernatants from autologous PBMCs, from the same donor from whom the VZV-specific CD8^+^ T cells were derived, co-cultured with uninfected- and VZV-infected HFLs were analyzed for IFNγ levels and virtually no IFNγ secretions were detected during co-culturing with uninfected- or VZV-infected HFLs (S7 Fig) indicating that only the VZV ORF34- and ORF18-specific CD8^+^ T cells were responsible for IFNγ inductions observed.

In addition, VZV ORF34- or ORF18-specific CD8^+^ T cells were co-cultured with HBVAFs in the presence of anti-PD-L1 blocking antibody (αPD-L1) or isotype controls for 24 h and cell culture supernatants were harvested and analyzed for IFNγ levels using ELISA. Neither VZV ORF34- nor ORF18-specific CD8^+^ T cells showed a change in IFNγ levels between isotype and αPD-L1 treatments when co-cultured with uninfected HBVAFs, whereas PD-L1 blockade in both ORF34- and ORF18-specific CD8^+^ T cells showed significant elevations in IFNγ levels as compared to isotype controls during co-culture with VZV-infected HBVAFs (P<0.001 and P<0.01, respectively) (S9 Fig).

In summary, blocking only PD-L1 enhanced CD8^+^ effector T cell functioning in response to VZV.

## Discussion

Herein, we examined the ability of VZV to infect human PBMCs and modulate expression of multiple immunoinhibitory proteins in a cell-type specific manner. Strikingly, VZV productively infected all PBMC subsets and we report here for the first time the productive infection of monocytes, B cells and NKT cells. While VZV has been widely regarded as a T cell tropic virus [1], a recent study showed that NK cells are productively infected by VZV and the most permissive to VZV infection when compared to T cells, NKT cells and CD3-CD56- lymphocytes [32]. While the infection rates amongst the lymphocyte populations reported here were similar to that of Campbell et al., their study did not investigate the permissiveness of VZV infection of monocytes or B cells and did not confirm productive infection of NKT cells or CD3-CD56- lymphocytes. We show here that all PBMC subsets are productively infected with VZV. This finding is consistent with the only other study investigating the permissiveness of VZV-infection of PBMCs during varicella and zoster in human patients, which revealed that T cells, monocytes and B cells express VZV gE [6]. Our results shed new light into the lymphotropism of VZV that leads to a better understanding of viral transmission during primary infection and reactivation.

During primary VZV infection, viral particles are inhaled and the first site of replication is tonsils where it is presumed that tonsillar T cells become infected and subsequently traffic virus to skin promoting the vesicular rash associated with varicella/chickenpox [1]. However, our studies suggest that additional immune cell types in peripheral blood are capable of being infected with VZV. Thus, the proximity of immune cells relative to the initial inhaled virus may be the determining factor for which immune cells are responsible for trafficking virus to skin and distal organs. In support of T cells being the “viral traffickers” of VZV to skin and distal organs, we report here that all T-lineage cells and NK cells have slight increases in surface VZV-gE expression at 48 and 72 hpi when compared to 24 hpi, while monocytes and B cells showed decreased surface VZV-gE expression over time. Therefore, T-lineage cells and NK cells appear to be more capable of withstanding viral infection for longer amounts of time that would enable them to traffic virus throughout the body. T-lineage cells are the most abundant immune cell in PBMCs (approximately 60–70%) while NK cells represent a much smaller fraction (<5%) which might explain why only T-lineage cells were initially recognized as carriers of virus during primary infection.

The preferential targeting of VZV infection towards innate immune cells (monocytes and NK cells) shown here would have several detrimental implications on the host immune response. First, an adaptive T cell or B cell immune response directed against VZV during primary infection takes approximately 2 weeks [33]. Therefore, VZV preferentially infects NK cells as these cells are capable of immediately detecting virus-infected cells as they use germline-encoded receptors to mount an immune response [34]. Indeed, fatal varicella has been identified in patients with NK cell deficiency [35, 36]. NKT cells are considered both an innate and adaptive immune cell and disseminated VZV infection due to VZV vaccine strain was reported in a child with a rare deficiency in NKT cells [37], indicating NKT cells contribute to host defense against VZV as well. Second, monocytes can differentiate into macrophages or dendritic cells (DCs), both of which are the main antigen presenting cells in the body required for CD4^+^ T cell activation. VZV infection of monocytes would presumably prevent monocyte maturation and subsequent CD4^+^ T cell activation that is absolutely required for controlling VZV infection along with simian varicella virus (SVV) [1, 38]. Interestingly, during primary infection of SVV in African green macaques, SVV was predominantly detected in both DCs and macrophages in the lung [39], while an additional study showed similar results in lymph nodes during SVV reactivation in rhesus macaques [40], all of which further support our findings.

A caveat to the primary infection model of SVV in African green macaques was that the SVV-GFP virus used in these studies was severely attenuated compared to wild-type virus with regards to viral DNA found in PBMCs (approximate 1000-fold decrease) [39]. We also show here that PBMCs infected with VZV-GFP have lower levels of infection across all PBMC subsets when compared to wild-type VZV.

Another striking finding was the induction of PD-L1 expression by VZV in all subsets of human PBMCs examined, along with the enhancement of T cell effector function after blocking PD-L1 in VZV-specific CD8^+^ T cells. The significant induction in IFNγ secretions during PD-L1 blockade in both VZV-specific CD8^+^ T cell lines shown here are similar to results in lymphocytic choriomeningitis virus (LCMV)-infected mice, where only anti-PD-L1 blockade and not anti-CTLA-4 rescued CD8^+^ T cell effector function [41]. We used flow cytometry gating based upon VZV gE expression to distinguish between VZV-infected and uninfected bystander cells. Bystander cells are presumably the CD8^+^ T cells responsible for killing VZV-infected cells as the infected VZV-specific CD8^+^ T cells probably do not elicit the same anti-viral immune response as their uninfected counterparts. Further supporting this notion, a bystander effect for PD-1 and CTLA-4 induction was only observed in VZV-specific CD8^+^ T cells and not in CD8^+^ T cells from healthy donor PBMCs which would contain <0.01% of VZV-specific CD8^+^ T cells as previously reported [33]. Therefore, utilizing flow cytometry gating we can explain why blocking PD-L1 and not CTLA-4 enhanced VZV-specific CD8^+^ T cell effector function. For example, both VZV-specific CD8^+^ T cell lines had modest 30–50% inductions in CTLA-4 and PD-1 expression levels in bystander cells compared to uninfected cells. However, VZV-infected PBMCs had a 2- to 14-fold induction in PD-L1 expression levels compared to uninfected PBMCs, which can explain the enhanced IFNγ levels observed when blocking PD-L1 and not CTLA-4. Induction of PD-L2 expression by VZV was minimal in PBMCs compared to PD-L1 levels, which can explain the lack of enhanced IFNγ levels observed when blocking PD-L2.

The induction of the PD-1:PD-L1 pathway during viral infection has been well documented and PD-1 expression has been shown to be increased in CD8^+^ T cells from zoster patients when compared to healthy controls [25]. We report here for the first time the VZV-mediated induction of PD-L1 expression in all VZV-infected PBMC subsets and VZV-specific CD8^+^ T cells that could clearly aid in immune suppression during zoster. VZV-infected PBMCs are present during both varicella and zoster but at a very low rate (1/10,000–100,000 cells) [6] which would provide minimal immune suppression. However, we found a bystander effect for induction of PD-L1 expression in both monocytes and CD4^+^ T cells, which may potentially amplify immune suppression during varicella or zoster especially considering that monocytes and CD4^+^ T cells represent the vast majority of immune cells in the PBMC pool (approximately 70–80%).

Aside from zoster, VZV reactivation can also produce VZV vasculopathy presenting as stroke, giant cell arteritis and granulomatosis aortitis [42]. Intriguingly, macrophages from patients with coronary artery disease have elevated PD-L1 expression and blocking PD-L1 in response to VZV enhanced VZV-specific CD4^+^ T cell responses [43]. Results from this study further support the role of VZV in promoting vascular disease and indicate a role of the PD-1:PD-L1 pathway during disease progression.

Overall, our comprehensive examination of VZV infection in human PBMCs shows that all immune cell populations are permissive for VZV infection. The ability of these immune cells to transmit virus combined with the induction of PD-L1 expression in all PBMCs provides novel immune evasion strategies elicited by VZV infection. Induction of PD-L1 in PBMCs combined with the induction of PD-1 expression on CD8^+^ T cells and NKT cells could greatly diminish the effectiveness of the immune system in clearing virus. The enhanced CD8^+^ T cell effector function after PD-L1 blockade during VZV infection shows that VZV-mediated immune suppression can be reversed to attenuate virus dissemination and multisystem disease associated with VZV.

## Materials and methods

### Cells and virus

Human fetal lung fibroblasts (HFLs) (ATCC, Manassas, VA) and human brain vascular adventitial fibroblasts (HBVAFs) (Sciencell, Carlsbad, CA) were cultured in basal fibroblast medium supplemented with 2% fetal bovine serum (FBS), 1% fibroblast growth serum and 1% 100X penicillin-streptomycin (PS) (Sciencell). Cells were grown to approximately 80% confluency and then infected with cell-associated viruses (40 pfu/ml) for 48–72 h. The VZV Ellen strain and vOka strains were used along with VZV-GFP that has been previously described [44]. At the height of infection culture medium was changed to basal fibroblast medium containing 2% FBS and 1% PS 4–6 h before immune cell addition. PBMCs isolated from whole blood using density gradient centrifugation on Ficoll (GE Healthcare Bio-Sciences, Pittsburgh, PA) were cultured in RPMI supplemented with 5% human serum (Gemini Bio-Products, West Sacramento, CA) and 1% PS (Sciencell) at a density of 2.0 x 10^6 cells/ml, after which 1.0 x 10^7 PBMCs were co-cultured for 48 h with uninfected- or VZV-infected HFLs grown in 10-cm^2^ cell culture dishes. There are 8.0 x 10^6 HFLs/HBVAFs on a confluent monolayer in 10-cm^2^ cell culture dishes, so approximately 1.25 PBMCs:1 HFL were co-cultured together. To confirm immunoinhibitory protein staining in flow cytometry, PBMCs were cultured alone with- or without treatment with PMA/Ionomycin (Biolegend, San Diego, CA) for 48 h before harvesting. Polyclonal VZV-specific CD8^+^ T cell lines that recognize specific epitopes in either VZV ORF34 or ORF18 proteins in the context of HLA A*0201 [30] were tetramer-sorted to >96% purity from PBMC of VZV-infected HLA A*0201-bearing donors and expanded with non-specific mitogens, cryopreserved, thawed before use, and cultured as described above in 12-well plates. Both VZV-specific CD8^+^ T cell lines were obtained from the same donor, such that autologous PBMCs were used as antigen presenting cells in some experiments. Polyclonal HSV-2-specific CD8^+^ T cell lines that recognize specific epitopes in either HSV-2 UL47 or UL49 proteins in the context of HLA-A*0201 (UL47) [45] and HLA-B*0702 (UL49) [45] were tetramer-sorted to >96% purity from PBMC of HSV-2-infected HLA A*0201- or HLA-B*0702-bearing donors and expanded with non-specific mitogens, cryopreserved, thawed before use, and cultured as described above in 12-well plates.

### Ethics statement

Frozen PBMCs from six healthy donors of age 29–50 years were obtained commercially from Astarte Biologics (Bothell, WA). Whole blood from twelve healthy donors of age 22–40 years were obtained commercially from Bonfils (Denver, CO). Informed consent was obtained by the source companies identified above from all adult subjects providing PBMCs and whole blood for this research project; samples were de-identified by the company prior to our receipt.

### Antibodies

For flow cytometry and FACS sorting experiments, fluorochrome-conjugated antibodies against the following antigens were used: CD45 (clone HI30; conjugated to BV421), CD3 (OKT3; BV605 & AF700), CD56 (HCD56; PE/Cy7), CD8 (SK1; BV510 & APC/Cy7), CD4 (SK3; FITC), CD19 (HIB19, PerCP/Cy5.5), CD14 (M5E2; APC), HLA-DR (LN3; BV711), PD-L1 (29E.2A3; BV605), LAG-3 (11C3C65; AF647), TIM-3 (F38-2E2; FITC & BV785), HLA-ABC (W6/32; PE/Cy7), Isotype (eBMG2b; PE) (MOPC-173; PE/Cy7) (all Biolegend); CD4 (SK3; UV395) and PD-L2 (MIH18; BV711 & BV785) (all BD Biosciences); CTLA-4 (14D3; PE/Cy7), PD-L1 (MIH1; APC), PD-1 (MIH4; FITC) (all ThermoFIsher, Waltham, MA); VZV gE (MilliporeSigma, Burlington, MA) conjugated in house to R-PE as previously described [31]. For immunofluorescence analyses experiments, unconjugated antibodies against the following antigens were used: VZV gB (Mouse: Abcam, Cambridge, MA) and VZV ORF63 (Rabbit) that was previously described [46]. Secondary antibodies consisted of Alexa Fluor 488 donkey anti-rabbit IgG and Alexa Fluor 594 donkey anti-mouse IgG (MilliporeSigma). Tetramers of HLA A*0201 and VZV ORF34 and ORF18 proteins were obtained from the Fred Hutchinson Cancer Research Center Immune Monitoring Core.

### Flow cytometry (FACS)

PBMCs or VZV-specific CD8^+^ T cells were harvested, washed with cold PBS and live/dead aqua-stained on ice as per the manufacturer’s protocol (ThermoFisher). Cells were then resuspended in FACS buffer (phosphate-buffered saline containing 1% FBS and 10 mm EDTA) with addition of antibodies on ice for at least 30 min. Then, cells were fixed in 1% paraformaldehyde (ThermoFisher) or fixed and permeabilized to stain for intracellular CTLA-4, using fixation/permeabilization kit (ThermoFisher). For FACS staining of PBMCs, after live/dead staining and washing, cells were cultured with human TruStain FcX (Biolegend) and True-Stain Monocyte Blocker (Biolegend) for 5 min on ice per manufacturer’s instructions before the addition of fluorescence-conjugated antibodies. Fluorescence-minus-one (FMO) controls were used for all immunoinhibitory proteins as were isotype controls for VZV gE and CTLA-4 staining. For flow cytometry staining of HBVAFs, cells were harvested with a citrate buffer as previously described [31] and then stained as described above. Cells were analyzed using an LSR-II flow cytometer (BD Immunocytometry Systems, San Jose, CA); >500,000 events were collected for all samples except for HBVAFs staining where 20,000 events were collected. Electronic compensation was performed with antibody capture beads (BD Biosciences, San Jose, CA) and subsequent data were analyzed using Diva software (BD Biosciences) and FlowJo software (Tree Star, Ashland, OR).

### FACS sorting

Human PBMCs were harvested on ice, washed with FACS buffer and then resuspended in FACS buffer with the addition of antibodies for 30 min on ice. No viability stain was conducted on the sorted cells, however, PBMCs were stained for live/dead aqua as described above before the sorting process to confirm viable cells (All PBMCs sorted were >90% viable). PBMCs were sorted using a FACS-Aria cytometer (BD Immunocytometry Systems). All sorted immune cell subsets were >93% pure based upon flow cytometry analyses. VZV-infected PBMCs were sorted based upon VZV-gE+ along with their lineage markers described in S1 Fig, while uninfected PBMCs were sorted based upon their lineage markers. A minimum of 15,000 sorted cells was obtained for all immune cell populations analyzed in all experiments. The addition of CD45 fluorochrome-conjugated antibody was used in FACS sorting gating scheme described in S1 Fig.

### Productive infection assays

After FACS sorting of PBMCs, individual VZV-infected immune cell subsets (15,000–20,000 per subset) were resuspended in complete RPMI media and added to semi-confluent monolayers of HFLs in 96-well plates. After 3 days of co-culturing, HFLs were washed with PBS and harvested using trypsin (Sciencell) then plated on ibidi 24-well μ-Plate (ibidi, Martinsried, Germany) for an additional 48 h to allow viral infection to spread. In some experiments, sorted PBMCs were washed in citrate buffer (40 mM C6H507Na3, 135 mM KCl, pH = 3.5) for 3 min, then washed with FACS buffer before co-culturing with HFLs to confirm no potential virus was “stuck” to PBMCs, ensuring that productive viral transmission occurred from VZV-infected PBMCs as previously described [29, 32]. For flow cytometry analyses of productively infected HFLs, HFLs were harvested 5 days after co-culturing as described above, followed by cell surface staining with VZV gE (R-PE) for 30 min on ice. HFLs were then fixed with 1% paraformaldehyde and analyzed using flow cytometry as described above.

### Immunofluorescence (IFA)

HFLs were propagated as described above in an ibidi 24-well μ-Plate (ibidi) and analyzed for immunofluorescence as previously described [47]. Briefly, HFLs were fixed with 4% paraformaldehyde for 20 min at room temperature, followed by permeabilization with Triton-X (0.1%) for 10 min and blocked with normal donkey serum (10%) for 1 hour, then stained against ORF63 (1:1000), VZV gB (1:500) or respective isotype control for 16 h at 4°C, while secondary antibodies were incubated for 1 hour at room temperature. Cells were washed 3 times in PBS following each antibody incubation. Following secondary antibody application and PBS washes, DAPI ([4′,6-]diamidino-2-phenylindole) (Vector Laboratories, Burlingame, CA) was added to the ibidi chambers at a 1:500 dilution for 5 min, washed in PBS and visualized by microscopy. For IFA of PBMCs, individual immune cell subsets were fixed in 1% paraformaldehyde for 24 h at 4°C after flow cytometry sorting and then cytospin was performed based upon manufacturer’s instructions (ThermoFisher). Briefly, cells were cytospinned at 800 RPM for 10 min, followed by permeabilization with Triton-X (0.1%) for 10 min and then blocked in normal donkey serum and stained for ORF63 and DAPI as described above. As a positive control for nuclear ORF63 expression and cellular size, uninfected- and VZV-infected HFLs were harvested with trypsin then fixed with 1% paraformaldehyde as described above and cytospinned in the same manner as PBMCs described above.

### Quantitative Reverse-Transcriptase PCR (q-RT-PCR) analyses

Total RNA was extracted from sorted uninfected- and VZV-infected human PBMCs along with from uninfected- and VZV-infected HFLs using the Direct-zol RNA miniprep kit (Zymo Research, Irvine, California). Enzyme degradation of residual DNA was completed using the Turbo-DNA free kit (ThermoFisher). First strand cDNA synthesis was completed using the Transcriptor first strand cDNA synthesis kit (Roche, San Francisco, California). RNA extraction, DNase treatment and cDNA synthesis were all performed according to the respective manufacturer’s instructions. For q-RT-PCR amplification, primer and probe sets for VZV ORF63 and ORF68 were used as previously described [48]; along with housekeeping gene glyceraldehyde 3-phosphate dehydrogenase (GAPdH; FWD: CACATGGCCTCCAAGGAGTAA, REV: TGAGGGTCTCTCTCTTCCTCTTGT, Probe: VIC/CTGGACCACCAGCCCCAGCAAG). Q-RT-PCR cycling conditions consisted of a holding stage at 95°C for 10 min, followed by a cycling stage of 95°C for 30 seconds and 60°C for 1 min (40 cycles). As a control for viral DNA contamination, cDNA synthesis was performed without the addition of reverse transcriptase and analyzed for q-RT-PCR analyses in the same manner as mentioned above.

### Blockade of PD-L1, PD-L2 and CTLA-4

For PD-L1 blockade experiments in HBVAFs, HLA A*0201 (+) HBVAFs were grown in 12-well plates at an approximate density of 5.0 x 10^5 cells/well, uninfected- or VZV-infected, treated with anti-PD-L1 (10 μg/ml; Biolegend clone 29E.2A3) or isotype control (10 μg/ml; Biolegend) for 30 min, and co-cultured with VZV-specific CD8^+^ T cells (2.0 x 10^6 cells/well) for 24 h before harvest of cell culture supernatants for IFNγ ELISA analyses. For PD-L1, PD-L2 and CTLA-4 blockade experiments using PBMCs from the same donor from whom the VZV-specific CD8^+^ T cells were derived, frozen PBMCs were co-cultured with uninfected- or VZV-infected HFLs for 48 h as described above and only non-adherent cells were harvested, washed in FACS buffer, seeded in 96-well plates (1.0 x 10^5 cells/well) and treated with either anti-PD-L1 (10 μg/ml; Biolegend), anti-PD-L2 (10 μg/ml; Biolegend clone MIH18), anti-CTLA-4 (10 μg/ml; Biolegend clone L3D10) or isotype control (10 μg/ml; Biolegend) for 1 h before co-culture with VZV-specific CD8^+^ T cells (1.0x10^5 cells/well) for 24 h. Cell culture supernatants were then harvested for IFNγ ELISA analyses.

### IFNγ ELISA

Levels of IFNγ in cell culture supernatants were measured using the Meso Scale Discovery human IFNγ kit (Rockville, MD) according to the manufacturer’s instructions. IFNγ levels were calculated by reference to a standard curve and all samples were analyzed in duplicate.

### Statistical analysis

Statistical analysis was performed using GraphPad Prism (GraphPad, San Diego, CA). Statistical significance was determined using the Student’s unpaired *t*-test, repeated measures (RM) one-way ANOVA with the Greenhouse-Geisse correction and Tukey posttest, and ordinary one-way ANOVA with the Tukey posttest.

## Supporting information

S1 Table Flow cytometry analyses of % VZV-gE+ immune cells from experiments described in Fig 1B using VZV Ellen strain. (DOCX)

S2 Table Flow cytometry analyses of % VZV-gE+ immune cells from experiments described in Fig 1C using vOka strain. (DOCX)

S3 Table Flow cytometry analyses of % VZV-GFP+ immune cells from experiments described in S2 Fig. (DOCX)

S4 Table Flow cytometry analyses of % VZV-gE+ immune cells at 24, 48 and 72 h post infection (hpi) using the VZV Ellen Strain. (DOCX)

S5 Table Q-RT-PCR analyses Ct values in uninfected- and VZV-infected monocytes, NK cells, NKT cells, B cells, CD4^+^ T cells, CD8^+^ T cells and HFLs from Fig 3. (DOCX)

S6 Table Average fold-change in MFI for immunoinhibitory proteins in VZV+ (V+), VZV-negative bystander (Bys) and uninfected (UI) monocytes, B cells, NK and NKT cells. (DOCX)

S7 Table Average fold-change in MFI for immunoinhibitory proteins in VZV+ (V+), VZV-negative bystander (Bys) and uninfected (UI) CD4^+^ T cells and CD8^+^ T cells. (DOCX)

S8 Table Average fold-change in MFI for immunoinhibitory proteins in VZV+ (V+), VZV-negative bystander (Bys) and uninfected (UI) VZV ORF18- or ORF34-specific CD8^+^ T cells. (DOCX)

S1 Fig Flow cytometry gating scheme for PBMC populations. After 48-h co-culture of PBMCs with uninfected- or VZV-infected HFLs, cells were harvested on ice, washed with PBS and stained using live/dead aqua followed by cell surface staining before flow cytometry analyses. Flow cytometry gating scheme, were sequentially gated by singlets, FSC/SSC for size, and gated for live/dead aqua-negative (live lymphocytes), followed by cell surface staining for CD3, CD56, CD19, CD14, CD4, CD8 and HLA-DR. NK = CD3^-^CD56^+^, NKT = CD3^+^CD56^+^, B cells = CD3^-^CD56^-^CD19^+^HLA-DR^+^, CD4^+^ T cell = CD56^-^CD3^+^CD4^+^CD8^-^, CD8^+^ T cell = CD56^-^CD3^+^CD8^+^CD4^-^. Live myeloid cells monocytes = CD3^-^CD56^-^CD19^-^CD14hi,HLA-DR^+^. FSC = forward scatter and SSC = side scatter.
(TIF)

S2 Fig VZV-GFP infection of human monocytes, B cells, NK cells, NKT cells, CD4^+^ T cells and CD8^+^ T cells. Human PBMCs were co-cultured with uninfected- (UI) or VZV-GFP-infected HFLs for 48 h then harvested and analyzed using flow cytometry. **(A)** Representative flow cytometry plots of live monocytes, NK cells, NKT cells, B cells, CD4^+^ T cells and CD8^+^ T cells, examining GFP expression. **(B)** Frequency of live GFP+ monocytes, NK cells, NKT cells, B cells, CD4^+^ T cells and CD8^+^ T cells from 5 healthy donors with bar graphs representing average % VZV-GFP+ cells ± SD. *P<0.05, **P<0.01; # above monocytes represents P<0.01 for significant increases in % VZV-GFP+ monocytes compared to all other immune cell populations analyzed. Statistical significance was determined using RM one-way ANOVA with the Greenhouse-Geisser correction and Tukey posttest.
(TIF)

S3 Fig Time course of VZV infection of human monocytes, B cells, NK cells, NKT cells, CD4^+^ T cells and CD8^+^ T cells. Human PBMCs were co-cultured with uninfected- (UI) or VZV-infected HFLs (Ellen strain) for 24, 48 and 72 h then harvested and analyzed using flow cytometry. Bar graphs represent average % VZV-gE+ immune cells ± SD. *P<0.05 and **P<0.01 for significant decreases in % VZV-gE+ immune cells compared to various time points analyzed. Results representative of 4 independent experiments using PBMCs from 4 different healthy controls. Statistical significance was determined using RM one-way ANOVA with the Greenhouse-Geisser correction and Tukey posttest.
(TIF)

S4 Fig Human monocytes, B cells and VZV ORF34- and ORF18-specific CD8^+^ T cells express higher levels of VZV gE than other PBMC subsets. Human PBMCs, VZV ORF34- or ORF18-specific CD8^+^ T cells were co-cultured with uninfected (UI) or VZV-infected HFLs for 48 h then harvested and analyzed using flow cytometry. **(A)** Representative flow cytometry gating scheme for VZV gE low expressing cells (Log0-1 for VZV gE expression, V+lo) and VZV gE high expressing cells (Log>1 for VZV gE expression, V+hi). **(B)** Summary of % VZV gE+hi cells in monocytes, B cells, NK cells, NKT cells, CD8^+^ T cells and CD4^+^ T cells. **(C)** Summary of % VZV gE+hi cells in VZV ORF34- or ORF18-specific CD8^+^ T cells compared to CD8^+^ T cells from human PBMCs. *P<0.05, **P<0.01, ***P<0.001, ****P<0.0001; # above monocytes represents P<0.01 for significant increases in % VZV gE+hi cells compared to all other immune cell populations analyzed except for B cells which was not significant. Statistical significance was determined using RM one-way ANOVA with the Greenhouse-Geisser correction and Tukey posttest.
(TIF)

S5 Fig Monocytes, NK cells, NKT cells, B cells, CD4^+^ T cells and CD8^+^ T cells are productively infected by VZV and capable of transmitting virus. Human PBMCs were co-cultured with VZV-infected HFLs for 48 h, then VZV-infected monocytes, NK, NKT, B cells, CD4^+^ T and CD8^+^ T cells were sorted using flow cytometry. Individual sorted immune cells were then co-cultured with uninfected HFLs. After 5 days of co-culture, flow cytometry analyses of VZV gE expression in HFLs revealed productive infection of HFLs by all individual immune cell populations analyzed. Negative controls were provided by isotype staining of VZV-infected HFLs. Results representative of PBMCs from 2 healthy donors.
(TIF)

S6 Fig Confirmation of immunoinhibitory protein induction in CD8^+^ T cells from human PBMCs treated with PMA/Ionomycin. Human PBMCs were cultured alone or in the presence of PMA/Ionomycin for 48 h then harvested and analyzed for PD-1, PD-L1, PD-L2, CTLA-4, LAG-3 and TIM-3 expression in live CD3^+^CD8^+^ T cells. Black histograms represent untreated PBMCs and red histograms represent PMA/Ionomycin treated PBMCs. Results representative of duplicate experiments from 2 healthy individual PBMCs.
(TIF)

S7 Fig Only VZV-specific CD8^+^ T cells induce IFNγ secretions when co-cultured with VZV-infected HBVAFs. HLA-A*0201^+^ HBVAFs were uninfected- (UI) or VZV-infected (VZV) for 72 h and then cultured alone or co-cultured with either HLA-A*0201-restricted VZV ORF18 specific CD8^+^ T cells (ORF18), or HLA-A*0201-restricted HSV-2 UL47-specific CD8^+^ T cells (UL47) or HLA-B*0702-restricted HSV-2 UL49-specific CD8^+^ T cells (UL49) for 24 h. In addition, autologous PBMCs from the same donor from whom the ORF18 CD8^+^ T cells (PBMC) were derived were co-cultured for 24 h with UI- or VZV-infected HBVAFs. Then cell culture supernatants were harvested and analyzed for IFNγ levels using ELISA. Results are representative of 3 independent experiments with bar graphs representing average IFNγ levels (pg/ml) ± SD. # represents P<0.0001 for significant inductions of IFNγ compared to all other experimental conditions. Statistical significance was determined using ordinary one-way ANOVA with the Tukey posttest.
(TIF)

S8 Fig VZV-infected HBVAFs have down-regulated MHC-I expression. HBVAFs were uninfected or VZV-infected (Ellen strain) for 72 h then harvested and analyzed for VZV gE and MHC-I expression using flow cytometry. **(A)** Representative flow cytometry plots of VZV gE and MHC-I expression in VZV-infected HBVAFs stained with isotype for MHC-I (Iso), uninfected HBVAFs (UI) or VZV-infected HBVAFs. VZV gE negative bystander cells (Bys) or VZV gE positive cells (V+). **(B)** Representative flow cytometry plots for MHC-I mean fluorescence intensity (MFI) expression levels in HBVAFs. Iso = black histograms, UI = grey histograms, Bys = blue histograms and V+ = Red histograms. **(C)** Summary of MHC-I MFI expression levels in UI, Bys, V+ and Iso stained HBVAFs. Results are representative of three independent experiments. Bar graphs represent average MHC-I MFI levels ± SD. **P<0.01 and ****P<0.0001. # represents P<0.0001 for significant increases in MHC-I MFI levels in UI, Bys and V+ HBVAFs compared to Iso stained HBVAFs. Statistical significance was determined using ordinary one-way ANOVA with Tukey posttest.
(TIF)

S9 Fig PD-L1 blockade enhances IFNγ secretion by VZV ORF34- or ORF18-specific CD8^+^ T cells during co-culture with HBVAFs. VZV ORF34- or ORF18-specific CD8^+^ T cells were co-cultured with uninfected- or VZV-infected HLA-A*0201^+^ HBVAFs for 24 h in the presence of PD-L1 blocking antibody (αPD-L1) or isotype controls. Then cell culture supernatants were harvested and analyzed for IFNγ levels using ELISA. Results are representative of 3 independent experiments with bar graphs representing average IFNγ levels (pg/ml) ± SD. **P<0.01, ***P<0.001. NS = not significant. Statistical significance was determined using RM one-way ANOVA with the Greenhouse-Geisser correction and Tukey posttest.
(TIF)
