# Supplementary material for: Varicella zoster virus productively infects human peripheral blood mononuclear cells to modulate expression of immunoinhibitory proteins and blocking PD-L1 enhances virus-specific CD8+ T cell effector function
Source: PLoS Pathog. 2019 Mar 14;15(3):e1007650. doi: 10.1371/journal.ppat.1007650 (PMC6435197; doi:10.1371/journal.ppat.1007650)
Supplement: S1 Table — (DOCX) [file ppat.1007650.s001.docx]

**S1 Table. Flow cytometry analyses of % VZV-gE+ immune cells from experiments described in Fig 1B using VZV Ellen strain.**

|  | **Monocyte** | **NK** | **NKT** | **B cell** | **CD4^+^ T** | **CD8^+^ T** |
| --- | --- | --- | --- | --- | --- | --- |
| **% VZV-gE+**  **Ellen Strain** | 69.96 ±5.86 | 31.29 ±3.44 | 19.38 ±6.59 | 15.77 ±2.05 | 14.25 ±1.93 | 9.84 ±1.22 |
| ***P* value vs. NK** | 0.001 | NA | NA | NA | NA | NA |
| ***P* value vs. NKT** | <0.0001 | 0.008 | NA | NA | NA | NA |
| ***P* value vs. B cell** | <0.0001 | 0.002 | 0.57 | NA | NA | NA |
| ***P* value vs. CD4+ T** | <0.0001 | 0.0004 | 0.03 | 0.99 | NA | NA |
| ***P* value vs. CD8+ T** | <0.0001 | 0.0002 | <0.0001 | 0.05 | 0.01 | NA |

Mean % VZV-gE+ cells ± SEM from 12 different healthy donor PBMC infections. *P* values were determined using RM one-way ANOVA with the Greenhouse-Geisser correction and Tukey posttest.
