# Supplementary material for: Varicella zoster virus productively infects human peripheral blood mononuclear cells to modulate expression of immunoinhibitory proteins and blocking PD-L1 enhances virus-specific CD8+ T cell effector function
Source: PLoS Pathog. 2019 Mar 14;15(3):e1007650. doi: 10.1371/journal.ppat.1007650 (PMC6435197; doi:10.1371/journal.ppat.1007650)
Supplement: S2 Table — (DOCX) [file ppat.1007650.s002.docx]

**S2 Table. Flow cytometry analyses of % VZV-gE+ immune cells from experiments described in Fig. 1C using vOka strain.**

|  | **Monocyte** | **NK** | **NKT** | **B cell** | **CD4^+^ T** | **CD8^+^ T** |
| --- | --- | --- | --- | --- | --- | --- |
| **% VZV-gE+**  **vOka** | 66.02 ±9.18 | 29.77 ±4.93 | 20.37 ±3.98 | 19.97 ±5.76 | 10.66 ±5.07 | 7.42 ±2.99 |
| ***P* value vs. NK** | 0.0005 | NA | NA | NA | NA | NA |
| ***P* value vs. NKT** | 0.0007 | 0.02 | NA | NA | NA | NA |
| ***P* value vs. B cell** | 0.0009 | 0.02 | 0.99 | NA | NA | NA |
| ***P* value vs. CD4+ T** | 0.0002 | 0.0005 | 0.06 | 0.12 | NA | NA |
| ***P* value vs. CD8+ T** | 0.0001 | <0.0001 | 0.002 | 0.008 | 0.26 | NA |

Mean % VZV-gE+ cells ± SEM from 5 different healthy donor PBMC infections. *P* values were determined using RM one-way ANOVA with the Greenhouse-Geisser correction and Tukey posttest.
