# Supplementary material for: Varicella zoster virus productively infects human peripheral blood mononuclear cells to modulate expression of immunoinhibitory proteins and blocking PD-L1 enhances virus-specific CD8+ T cell effector function
Source: PLoS Pathog. 2019 Mar 14;15(3):e1007650. doi: 10.1371/journal.ppat.1007650 (PMC6435197; doi:10.1371/journal.ppat.1007650)
Supplement: S3 Table — (DOCX) [file ppat.1007650.s003.docx]

**S3 Table. Flow cytometry analyses of % VZV-GFP+ immune cells from experiments described in S2 Fig.**

|  | **Monocyte** | **NK** | **NKT** | **B cell** | **CD4^+^ T** | **CD8^+^ T** |
| --- | --- | --- | --- | --- | --- | --- |
| **% VZV-GFP+** | 35.50 ±5.72 | 7.07 ±1.85 | 3.85 ±2.15 | 6.12 ±1.4 | 2.12 ±1.43 | 2.03 ±1.35 |
| ***P* value vs. NK** | 0.001 | NA | NA | NA | NA | NA |
| ***P* value vs. NKT** | 0.003 | 0.15 | NA | NA | NA | NA |
| ***P* value vs. B cell** | 0.004 | 0.97 | 0.53 | NA | NA | NA |
| ***P* value vs. CD4+ T** | 0.001 | 0.002 | 0.45 | 0.06 | NA | NA |
| ***P* value vs. CD8+ T** | 0.001 | 0.002 | 0.43 | 0.05 | 0.69 | NA |

Mean % VZV-GFP+ cells ± SD from 5 different healthy donor PBMC infections. *P* values were determined using RM one-way ANOVA with the Greenhouse-Geisser correction and Tukey posttest.
