# Supplementary material for: Varicella zoster virus productively infects human peripheral blood mononuclear cells to modulate expression of immunoinhibitory proteins and blocking PD-L1 enhances virus-specific CD8+ T cell effector function
Source: PLoS Pathog. 2019 Mar 14;15(3):e1007650. doi: 10.1371/journal.ppat.1007650 (PMC6435197; doi:10.1371/journal.ppat.1007650)
Supplement: S4 Table — (DOCX) [file ppat.1007650.s004.docx]

**S4 Table. Flow cytometry analyses of % VZV-gE+ immune cells at 24, 48 and 72 hours post infection (hpi) using the VZV Ellen strain.**

|  | **Monocyte** | **NK** | **NKT** | **B cell** | **CD4^+^ T** | **CD8^+^ T** |
| --- | --- | --- | --- | --- | --- | --- |
| **% VZV-gE+**  **Ellen Strain 24 hpi** | 87.5 ±4.76 | 19.0 ±2.65 | 10.7 ±2.6 | 30.4 ±12.8 | 9.1 ±2.2 | 5.6 ±1.0 |
| **% VZV-gE+**  **Ellen Strain 48 hpi** | 88.2 ±9.7 | 22.8 ±9.2 | 17.4 ±7.8 | 13.9 ±4.5 | 17.0 ±8.9 | 10.3 ±5.2 |
| **% VZV-gE+**  **Ellen Strain 72 hpi** | 71.3 ±11.7 | 21.9 ±10.3 | 17.3 ±5.9 | 7.8 ±2.9 | 16.9 ±6.1 | 10.6 ±2.6 |
| ***P* value 24 hpi vs. 48 hpi** | 0.98 | 0.78 | 0.46 | 0.04 | 0.30 | 0.26 |
| ***P* value 24 hpi vs. 72 hpi** | 0.11 | 0.88 | 0.30 | 0.008 | 0.20 | 0.10 |
| ***P* value 48 hpi vs. 72 hpi** | 0.006 | 0.94 | 0.99 | 0.56 | 0.99 | 0.99 |

Mean % VZV-gE+ cells ± SD from 4 different healthy donor PBMC infections. *P* values were determined using RM one-way ANOVA with the Greenhouse-Geisser correction and Tukey posttest.
