# Supplementary material for: Varicella zoster virus productively infects human peripheral blood mononuclear cells to modulate expression of immunoinhibitory proteins and blocking PD-L1 enhances virus-specific CD8+ T cell effector function
Source: PLoS Pathog. 2019 Mar 14;15(3):e1007650. doi: 10.1371/journal.ppat.1007650 (PMC6435197; doi:10.1371/journal.ppat.1007650)
Supplement: S5 Table — (DOCX) [file ppat.1007650.s005.docx]

**S5 Table. Q-RT-PCR analyses Ct values in uninfected- and VZV-infected monocytes, NK cells, NKT cells, B cells, CD4^+^ T cells, CD8^+^ T cells and HFLs from Figure 3.**

| **VZV-infected** | **Monocyte** | **NK** | **NKT** | **B cell** | **CD4^+^ T** | **CD8^+^ T** | **HFLs** |
| --- | --- | --- | --- | --- | --- | --- | --- |
| **GAPdH** | 29.4 ±2.6 | 30.3 ±2.4 | 32.9 ±1.0 | 30.3 ±1.8 | 27.7 ±1.4 | 30.8 ±1.5 | 24.4 ±0.4 |
| **VZV ORF63** | 20.3 ±3.5 | 24.4 ±2.9 | 28.0 ±1.1 | 21.8 ±3.4 | 23.3 ±3.1 | 26.2 ±2.0 | 24.7 ±0.5 |
| **VZV ORF68** | 29.7 ±4.5 | 30.8 ±0.8 | 34.7 ±0.6 | 30.4 ±4.1 | 31.2 ±2.7 | 33.6 ±1.6 | 32.4 ±1.3 |
| **GAPdH RT^-/-^** | N/A | N/A | N/A | N/A | N/A | N/A | N/A |
| **ORF63 RT^-/-^** | N/A | N/A | N/A | N/A | N/A | N/A | N/A |
|  |  |  |  |  |  |  |  |
| **Uninfected** | **Monocyte** | **NK** | **NKT** | **B cell** | **CD4^+^ T** | **CD8^+^ T** | **HFLs** |
| **GAPdH** | 27.2 ±1.4 | 28.3 ±0.9 | 28.2 ±1.2 | 27.2 ±0.6 | 25.6 ±0.6 | 26.3 ±0.6 | 21.4 ±0.6 |
| **VZV ORF63** | N/A | N/A | N/A | N/A | N/A | N/A | N/A |

Mean Ct value ± SD. N/A=Ct value was undetermined. RT^-/-^= Q-RT-PCR analyses without the addition of reverse transcriptase to the cDNA reaction to confirm no contamination from viral DNA.
