# Supplementary material for: Varicella zoster virus productively infects human peripheral blood mononuclear cells to modulate expression of immunoinhibitory proteins and blocking PD-L1 enhances virus-specific CD8+ T cell effector function
Source: PLoS Pathog. 2019 Mar 14;15(3):e1007650. doi: 10.1371/journal.ppat.1007650 (PMC6435197; doi:10.1371/journal.ppat.1007650)
Supplement: S6 Table — (DOCX) [file ppat.1007650.s006.docx]

**S6 Table. Average fold-change in MFI for immunoinhibitory protein expression in VZV+ (V+), VZV-negative bystander (Bys) and uninfected (UI) monocytes, B cells, NK and NKT cells from Fig. 5.**

|  | **Lin^-^CD14^+^HLA-DR^+^ Monocyte** | | | | | |
| --- | --- | --- | --- | --- | --- | --- |
|  | **Fold-change MFI ± SEM** | | | ***P* values** | | |
|  | **Bys/UI** | **V+/UI** | **V+/Bys** | **Bys/UI** | **V+/UI** | **V+/Bys** |
| **PD-L1** | 3.77 ±0.34 | 6.46 ±1.34 | 1.67 ±0.23 | 0.003 | <0.0001 | 0.03 |
| **PD-L2** | 1.07 ±0.19 | 1.35 ±0.25 | 1.24 ±0.02 | 0.97 | 0.51 | 0.64 |
| **PD-1** | 0.47 ±0.06 | 0.54 ±0.08 | 1.12 ±0.05 | 0.10 | 0.17 | 0.95 |

|  | **CD3^-^CD56^-^CD19^+^ B cell** | | | | | |
| --- | --- | --- | --- | --- | --- | --- |
|  | **Fold-change MFI ± SEM** | | | ***P* values** | | |
|  | **Bys/UI** | **V+/UI** | **V+/Bys** | **Bys/UI** | **V+/UI** | **V+/Bys** |
| **PD-L1** | 0.78 ±0.08 | 14.20 ±8.15 | 13.69 ±7.21 | 0.84 | 0.003 | 0.0008 |
| **PD-L2** | 2.11 ±0.17 | 2.42 ±0.19 | 1.15 ±0.02 | 0.07 | 0.02 | 0.79 |
| **PD-1** | 1.04 ±0.13 | 1.11 ±0.12 | 1.09 ±0.09 | 0.99 | 0.98 | 0.98 |

|  | **CD3^-^CD56^+^ NK cell** | | | | | |
| --- | --- | --- | --- | --- | --- | --- |
|  | **Fold-change MFI ± SEM** | | | ***P* values** | | |
|  | **Bys/UI** | **V+/UI** | **V+/Bys** | **Bys/UI** | **V+/UI** | **V+/Bys** |
| **PD-L1** | 1.31 ±0.14 | 6.64 ±2.10 | 4.88 ±1.40 | 0.09 | 0.01 | 0.02 |
| **PD-L2** | 1.23 ±0.23 | 1.70 ±0.22 | 1.95 ±0.49 | 0.12 | 0.19 | 0.06 |
| **PD-1** | 1.09 ±0.25 | 1.06 ±0.20 | 1.02 ±0.09 | 0.99 | 0.99 | 0.99 |

|  | **CD3^+^CD56^+^ NKT cell** | | | | | |
| --- | --- | --- | --- | --- | --- | --- |
|  | **Fold-change MFI ± SEM** | | | ***P* values** | | |
|  | **Bys/UI** | **V+/UI** | **V+/Bys** | **Bys/UI** | **V+/UI** | **V+/Bys** |
| **PD-L1** | 1.19 ±0.18 | 8.93 ±5.47 | 5.91 ±2.98 | 0.99 | 0.002 | 0.001 |
| **PD-L2** | 0.93 ±0.20 | 1.60 ±0.28 | 1.86 ±0.58 | 0.88 | 0.61 | 0.34 |
| **PD-1** | 1.51 ±0.15 | 3.01 ±0.53 | 1.99 ±0.29 | 0.67 | 0.005 | 0.04 |

Mean fold-change in MFI ± SEM. *P* values were determined using RM one-way ANOVA with the Greenhouse-Geisser correction and Tukey posttest.
