# Supplementary material for: Varicella zoster virus productively infects human peripheral blood mononuclear cells to modulate expression of immunoinhibitory proteins and blocking PD-L1 enhances virus-specific CD8+ T cell effector function
Source: PLoS Pathog. 2019 Mar 14;15(3):e1007650. doi: 10.1371/journal.ppat.1007650 (PMC6435197; doi:10.1371/journal.ppat.1007650)
Supplement: S7 Table — (DOCX) [file ppat.1007650.s007.docx]

**S7 Table. Average fold-change in MFI for immunoinhibitory protein expression in VZV+ (V+), VZV-negative bystander (Bys) and uninfected (UI) CD4^+^ T cells and CD8^+^ T cells from Fig 6.**

|  | **CD3^+^CD4^+^ T cell** | | | | | |
| --- | --- | --- | --- | --- | --- | --- |
|  | **Fold-change MFI ± SEM** | | | ***P* values** | | |
|  | **Bys/UI** | **V+/UI** | **V+/Bys** | **Bys/UI** | **V+/UI** | **V+/Bys** |
| **PD-L1** | 2.64 ±0.20 | 3.90 ±0.73 | 1.51 ±0.29 | 0.0007 | <0.0001 | 0.07 |
| **PD-L2** | 1.25 ±0.15 | 1.50 ±0.17 | 1.21 ±0.01 | 0.93 | 0.42 | 0.64 |
| **PD-1** | 0.93 ±0.04 | 1.26 ±0.05 | 1.35 ±0.03 | 0.85 | 0.46 | 0.21 |
| **CTLA-4** | 0.89 ±0.04 | 1.06 ±0.07 | 1.19 ±0.04 | 0.87 | 0.87 | 0.58 |
| **LAG-3** | 1.30 ±0.08 | 1.31 ±0.07 | 1.02 ±0.04 | 0.07 | 0.06 | 0.99 |
| **TIM-3** | 0.94 ±0.02 | 1.06 ±0.03 | 1.13 ±0.02 | 0.57 | 0.63 | 0.15 |

|  | **CD3^+^CD8^+^ T cell** | | | | | |
| --- | --- | --- | --- | --- | --- | --- |
|  | **Fold-change MFI ± SEM** | | | ***P* values** | | |
|  | **Bys/UI** | **V+/UI** | **V+/Bys** | **Bys/UI** | **V+/UI** | **V+/Bys** |
| **PD-L1** | 2.41 ±0.31 | 6.34 ±0.66 | 2.81 ±0.36 | 0.21 | <0.0001 | 0.001 |
| **PD-L2** | 1.20 ±0.11 | 1.52 ±0.13 | 1.27 ±0.01 | 0.98 | 0.48 | 0.57 |
| **PD-1** | 1.27 ±0.21 | 2.50 ±0.34 | 2.02 ±0.10 | 0.86 | 0.0002 | 0.0007 |
| **CTLA-4** | 0.92 ±0.02 | 1.11 ±0.03 | 1.20 ±0.01 | 0.72 | 0.44 | 0.13 |
| **LAG-3** | 1.28 ±0.13 | 1.59 ±0.15 | 1.24 ±0.05 | 0.50 | 0.09 | 0.53 |
| **TIM-3** | 0.95 ±0.02 | 1.08 ±0.04 | 1.13 ±0.03 | 0.60 | 0.40 | 0.08 |

Mean fold-change in MFI ± SEM. *P* values were determined using RM one-way ANOVA with the Greenhouse-Geisser correction and Tukey posttest.
