# Supplementary material for: Varicella zoster virus productively infects human peripheral blood mononuclear cells to modulate expression of immunoinhibitory proteins and blocking PD-L1 enhances virus-specific CD8+ T cell effector function
Source: PLoS Pathog. 2019 Mar 14;15(3):e1007650. doi: 10.1371/journal.ppat.1007650 (PMC6435197; doi:10.1371/journal.ppat.1007650)
Supplement: S8 Table — (DOCX) [file ppat.1007650.s008.docx]

**S8 Table. Average fold-change in MFI for immunoinhibitory protein expression in VZV+ (V+), VZV-negative bystander (Bys) and uninfected (UI) VZV ORF18- or ORF34-specific CD8^+^ T cells from Fig 8.**

|  | **VZV ORF18-specific CD8^+^ T cell** | | | | | |
| --- | --- | --- | --- | --- | --- | --- |
|  | **Fold-change MFI ± SEM** | | | ***P* values** | | |
|  | **Bys/UI** | **V+/UI** | **V+/Bys** | **Bys/UI** | **V+/UI** | **V+/Bys** |
| **PD-L1** | 1.39 ±0.14 | 4.73 ±0.65 | 3.87 ±0.65 | 0.10 | <0.0001 | <0.0001 |
| **PD-L2** | 1.32 ±0.18 | 3.93 ±0.52 | 3.53 ±0.72 | 0.11 | <0.0001 | <0.0001 |
| **PD-1** | 1.54 ±0.09 | 3.04 ±0.28 | 1.97 ±0.10 | 0.0008 | <0.0001 | <0.0001 |
| **CTLA-4** | 1.42 ±0.07 | 2.24 ±0.14 | 1.58 ±0.05 | 0.0005 | <0.0001 | <0.0001 |
| **LAG-3** | 1.13 ±0.10 | 1.15 ±0.23 | 1.00 ±0.18 | 0.37 | 0.35 | 0.56 |
| **TIM-3** | 0.91 ±0.08 | 1.01 ±0.12 | 1.16 ±0.13 | 0.99 | 0.98 | 0.99 |

|  | **VZV ORF34-specific CD8^+^ T cell** | | | | | |
| --- | --- | --- | --- | --- | --- | --- |
|  | **Fold-change MFI ± SEM** | | | ***P* values** | | |
|  | **Bys/UI** | **V+/UI** | **V+/Bys** | **Bys/UI** | **V+/UI** | **V+/Bys** |
| **PD-L1** | 1.26 ±0.11 | 5.57 ±1.39 | 4.94 ±1.37 | 0.08 | 0.002 | 0.004 |
| **PD-L2** | 1.25 ±0.10 | 3.52 ±0.33 | 2.92 ±0.33 | 0.06 | <0.0001 | 0.0002 |
| **PD-1** | 1.39 ±0.12 | 2.53 ±0.22 | 1.88 ±0.11 | 0.02 | <0.0001 | <0.0001 |
| **CTLA-4** | 1.32 ±0.05 | 2.23 ±0.21 | 1.68 ±0.12 | 0.003 | <0.0001 | <0.0001 |
| **LAG-3** | 0.89 ±0.12 | 1.07 ±0.22 | 1.08 ±0.21 | 0.37 | 0.35 | 0.56 |
| **TIM-3** | 1.05 ±0.13 | 1.18 ±0.13 | 1.26 ±0.24 | 0.66 | 0.10 | 0.94 |

Mean fold-change in MFI ± SEM. *P* values were determined using RM one-way ANOVA with the Greenhouse-Geisser correction and Tukey posttest.
