# Supplementary figures and images for: Varicella zoster virus productively infects human peripheral blood mononuclear cells to modulate expression of immunoinhibitory proteins and blocking PD-L1 enhances virus-specific CD8+ T cell effector function
Source: PLoS Pathog. 2019 Mar 14;15(3):e1007650. doi: 10.1371/journal.ppat.1007650 (PMC6435197; doi:10.1371/journal.ppat.1007650)

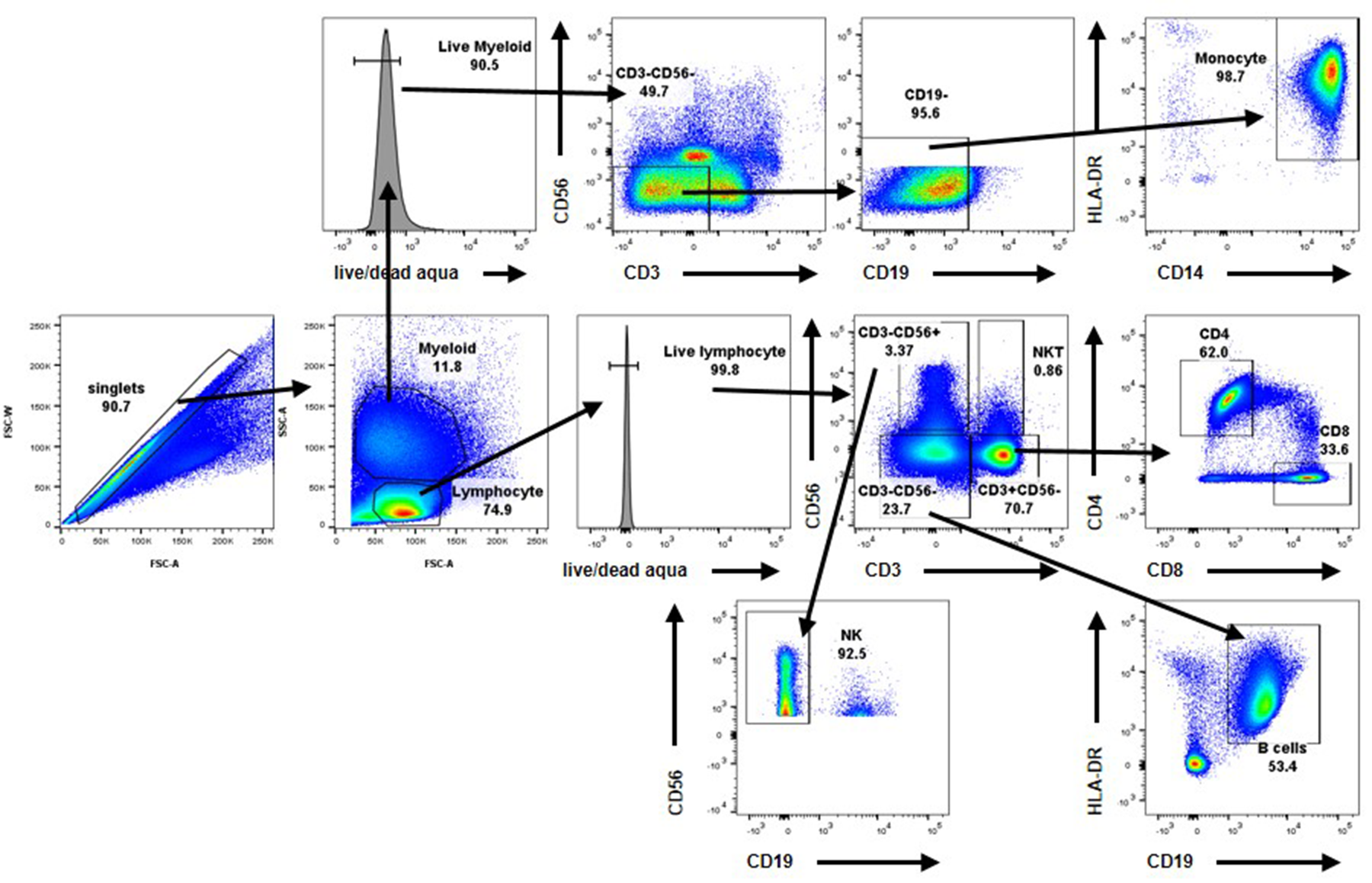

Supplement: S1 Fig — After 48-h co-culture of PBMCs with uninfected- or VZV-infected HFLs, cells were harvested on ice, washed with PBS and stained using live/dead aqua followed by cell surface staining before flow cytometry analyses. Flow cytometry gating scheme, were sequentially gated by singlets, FSC/SSC for size, and gated for live/dead aqua-negative (live lymphocytes), followed by cell surface staining for CD3, CD56, CD19, CD14, CD4, CD8 and HLA-DR. NK = CD3-CD56+, NKT = CD3+CD56+, B cells = CD3-CD56-CD19+HLA-DR+, CD4+ T cell = CD56-CD3+CD4+CD8-, CD8+ T cell = CD56-CD3+CD8+CD4-. Live myeloid cells monocytes = CD3-CD56-CD19-CD14hi,HLA-DR+. FSC = forward scatter and SSC = side scatter. (TIF) [file ppat.1007650.s009.tif]

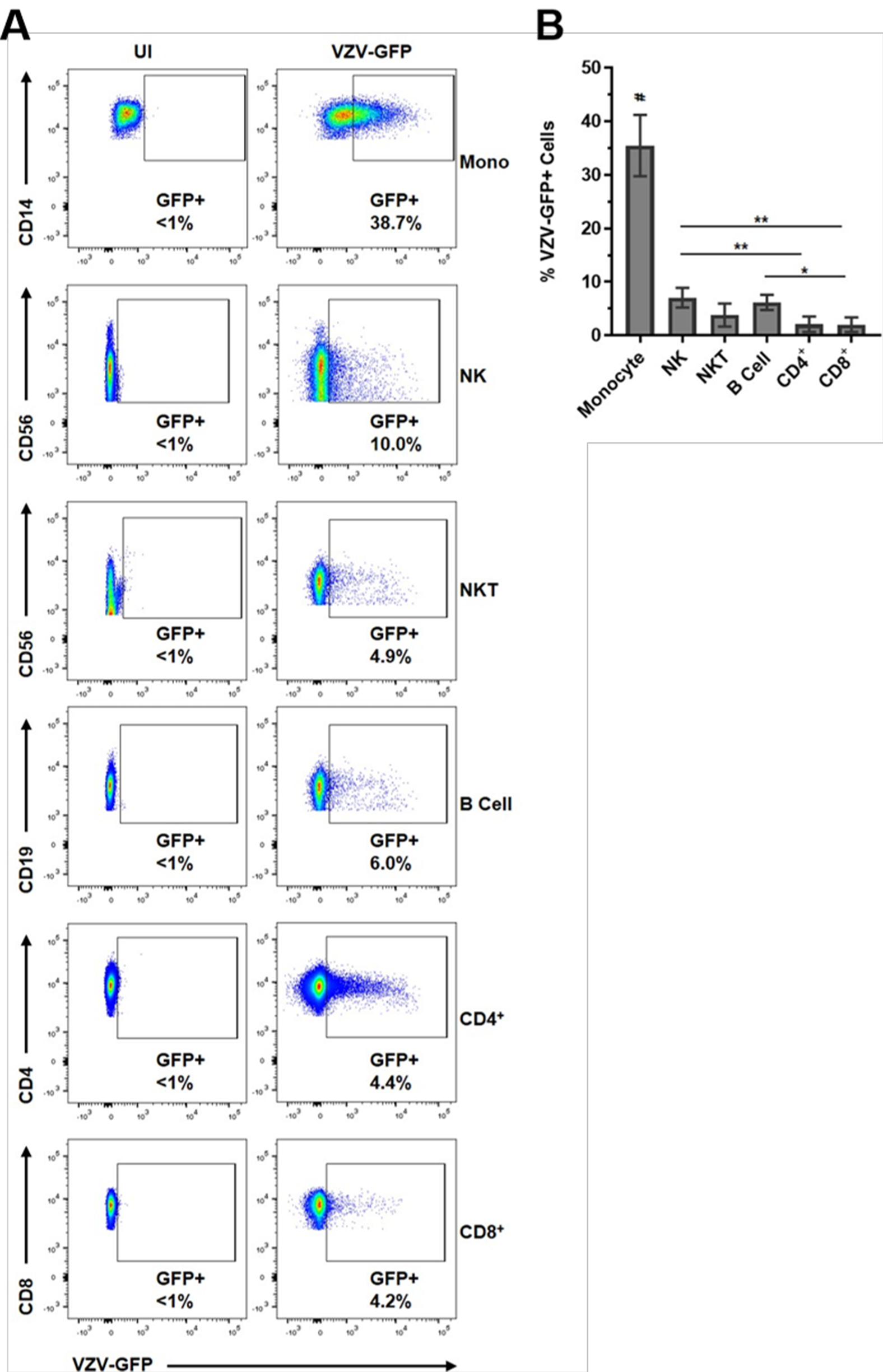

Supplement: S2 Fig — Human PBMCs were co-cultured with uninfected- (UI) or VZV-GFP-infected HFLs for 48 h then harvested and analyzed using flow cytometry. (A) Representative flow cytometry plots of live monocytes, NK cells, NKT cells, B cells, CD4+ T cells and CD8+ T cells, examining GFP expression. (B) Frequency of live GFP+ monocytes, NK cells, NKT cells, B cells, CD4+ T cells and CD8+ T cells from 5 healthy donors with bar graphs representing average % VZV-GFP+ cells ± SD. *P<0.05, **P<0.01; # above monocytes represents P<0.01 for significant increases in % VZV-GFP+ monocytes compared to all other immune cell populations analyzed. Statistical significance was determined using RM one-way ANOVA with the Greenhouse-Geisser correction and Tukey posttest. (TIF) [file ppat.1007650.s010.tif]

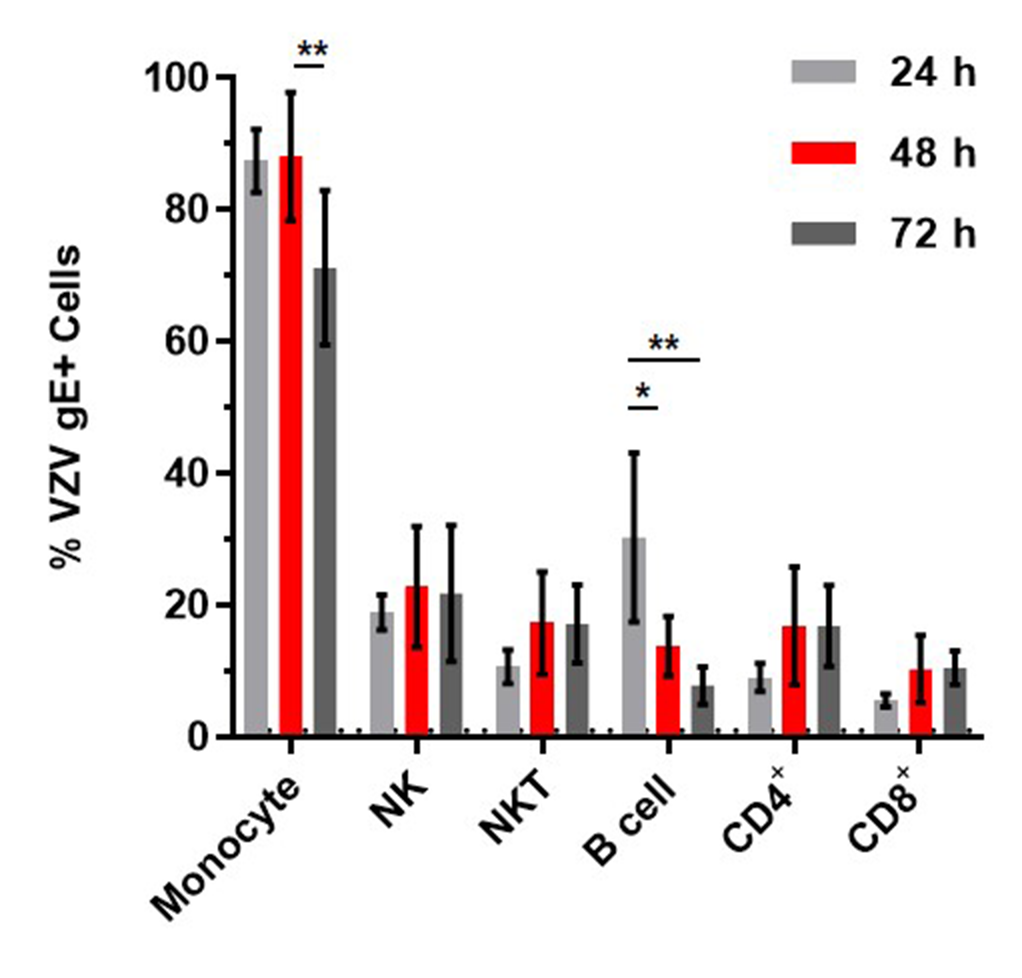

Supplement: S3 Fig — Human PBMCs were co-cultured with uninfected- (UI) or VZV-infected HFLs (Ellen strain) for 24, 48 and 72 h then harvested and analyzed using flow cytometry. Bar graphs represent average % VZV-gE+ immune cells ± SD. *P<0.05 and **P<0.01 for significant decreases in % VZV-gE+ immune cells compared to various time points analyzed. Results representative of 4 independent experiments using PBMCs from 4 different healthy controls. Statistical significance was determined using RM one-way ANOVA with the Greenhouse-Geisser correction and Tukey posttest. (TIF) [file ppat.1007650.s011.tif]

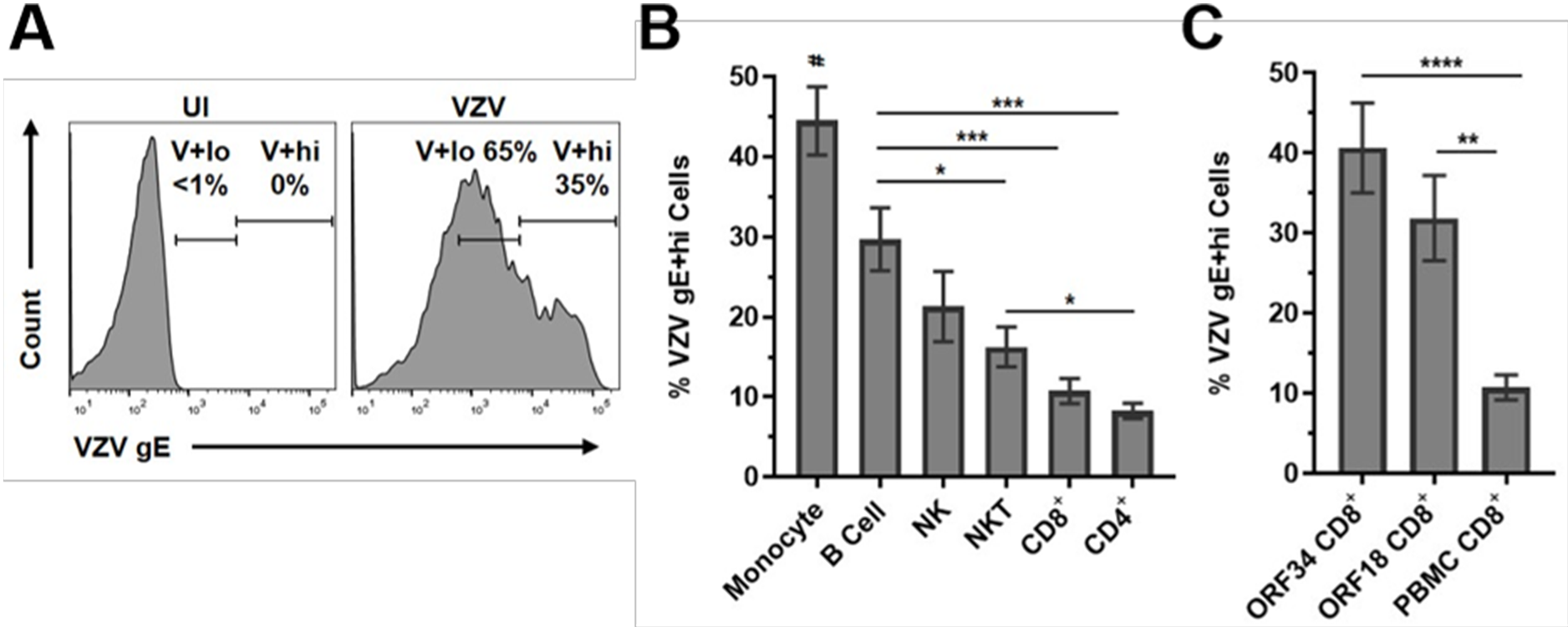

Supplement: S4 Fig — Human PBMCs, VZV ORF34- or ORF18-specific CD8+ T cells were co-cultured with uninfected (UI) or VZV-infected HFLs for 48 h then harvested and analyzed using flow cytometry. (A) Representative flow cytometry gating scheme for VZV gE low expressing cells (Log0-1 for VZV gE expression, V+lo) and VZV gE high expressing cells (Log>1 for VZV gE expression, V+hi). (B) Summary of % VZV gE+hi cells in monocytes, B cells, NK cells, NKT cells, CD8+ T cells and CD4+ T cells. (C) Summary of % VZV gE+hi cells in VZV ORF34- or ORF18-specific CD8+ T cells compared to CD8+ T cells from human PBMCs. *P<0.05, **P<0.01, ***P<0.001, ****P<0.0001; # above monocytes represents P<0.01 for significant increases in % VZV gE+hi cells compared to all other immune cell populations analyzed except for B cells which was not significant. Statistical significance was determined using RM one-way ANOVA with the Greenhouse-Geisser correction and Tukey posttest. (TIF) [file ppat.1007650.s012.tif]

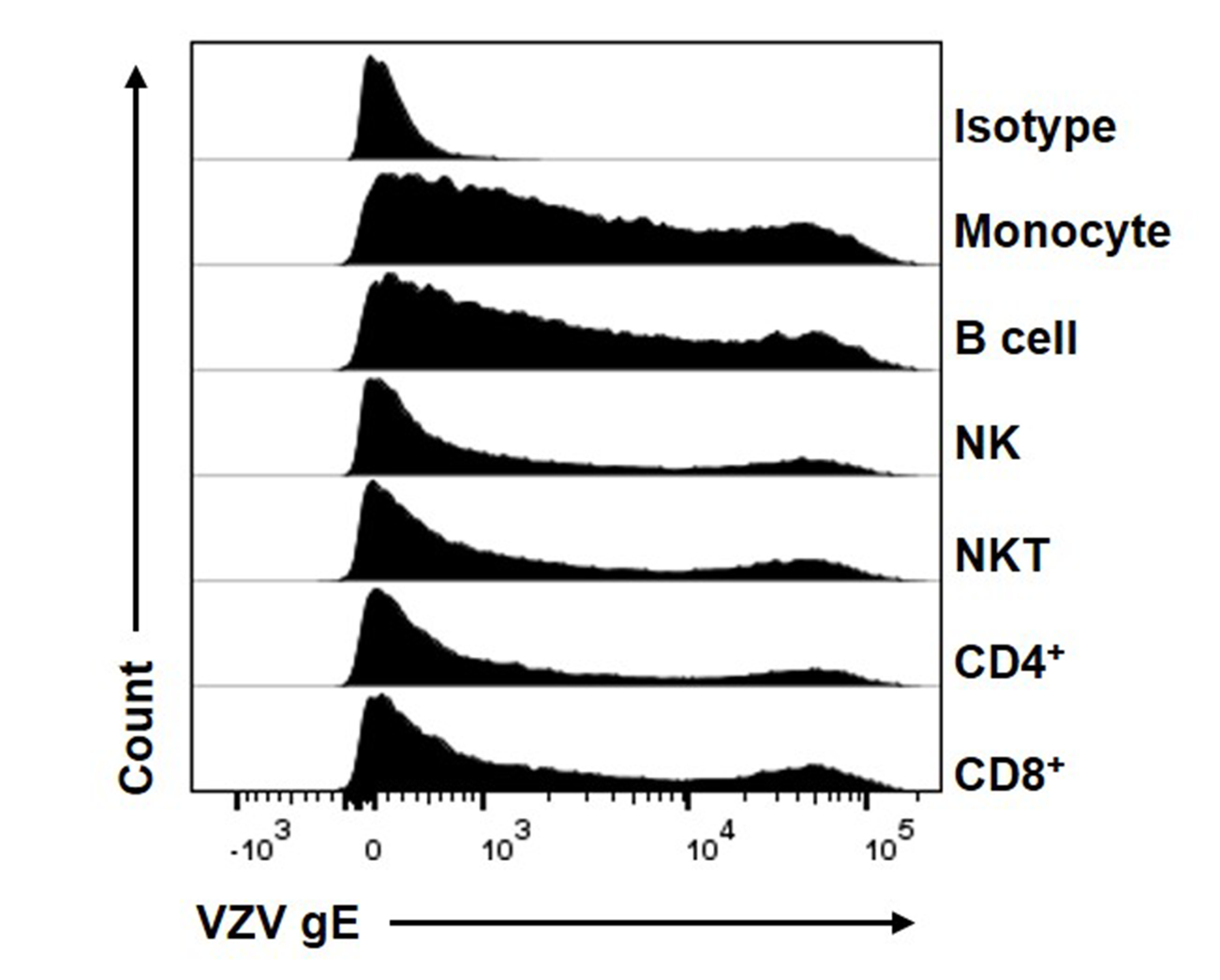

Supplement: S5 Fig — Human PBMCs were co-cultured with VZV-infected HFLs for 48 h, then VZV-infected monocytes, NK, NKT, B cells, CD4+ T and CD8+ T cells were sorted using flow cytometry. Individual sorted immune cells were then co-cultured with uninfected HFLs. After 5 days of co-culture, flow cytometry analyses of VZV gE expression in HFLs revealed productive infection of HFLs by all individual immune cell populations analyzed. Negative controls were provided by isotype staining of VZV-infected HFLs. Results representative of PBMCs from 2 healthy donors. (TIF) [file ppat.1007650.s013.tif]

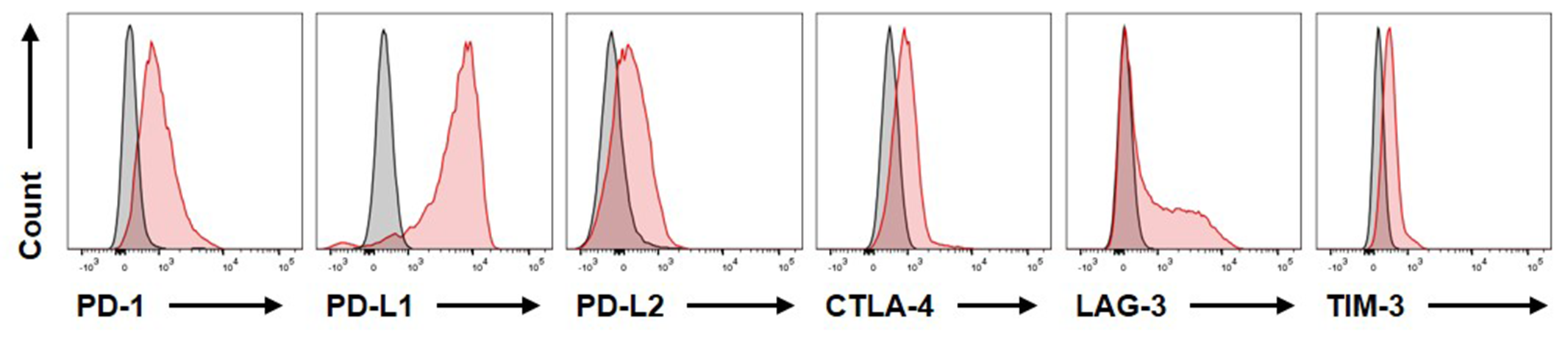

Supplement: S6 Fig — Human PBMCs were cultured alone or in the presence of PMA/Ionomycin for 48 h then harvested and analyzed for PD-1, PD-L1, PD-L2, CTLA-4, LAG-3 and TIM-3 expression in live CD3+CD8+ T cells. Black histograms represent untreated PBMCs and red histograms represent PMA/Ionomycin treated PBMCs. Results representative of duplicate experiments from 2 healthy individual PBMCs. (TIF) [file ppat.1007650.s014.tif]

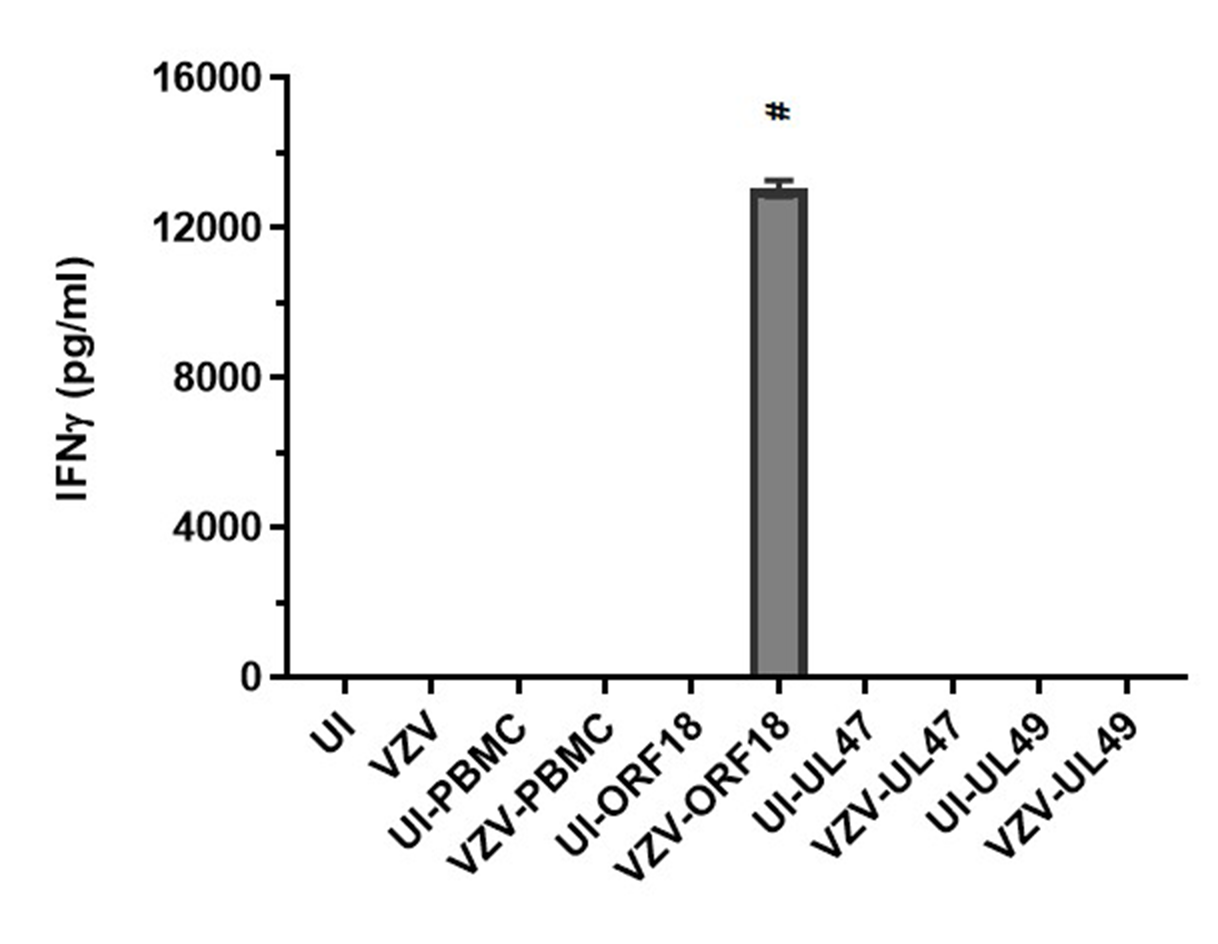

Supplement: S7 Fig — HLA-A*0201+ HBVAFs were uninfected- (UI) or VZV-infected (VZV) for 72 h and then cultured alone or co-cultured with either HLA-A*0201-restricted VZV ORF18 specific CD8+ T cells (ORF18), or HLA-A*0201-restricted HSV-2 UL47-specific CD8+ T cells (UL47) or HLA-B*0702-restricted HSV-2 UL49-specific CD8+ T cells (UL49) for 24 h. In addition, autologous PBMCs from the same donor from whom the ORF18 CD8+ T cells (PBMC) were derived were co-cultured for 24 h with UI- or VZV-infected HBVAFs. Then cell culture supernatants were harvested and analyzed for IFNγ levels using ELISA. Results are representative of 3 independent experiments with bar graphs representing average IFNγ levels (pg/ml) ± SD. # represents P<0.0001 for significant inductions of IFNγ compared to all other experimental conditions. Statistical significance was determined using ordinary one-way ANOVA with the Tukey posttest. (TIF) [file ppat.1007650.s015.tif]

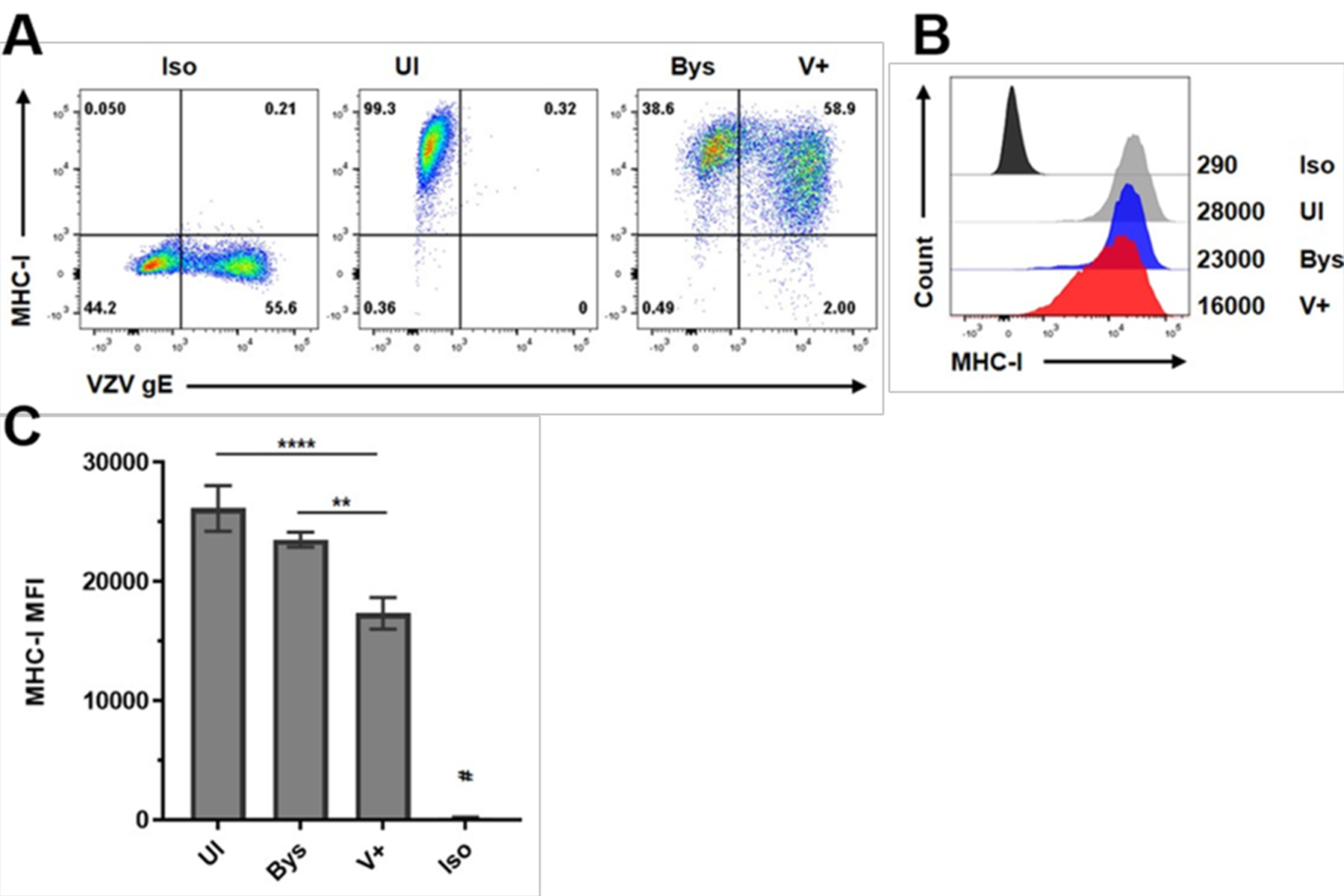

Supplement: S8 Fig — HBVAFs were uninfected or VZV-infected (Ellen strain) for 72 h then harvested and analyzed for VZV gE and MHC-I expression using flow cytometry. (A) Representative flow cytometry plots of VZV gE and MHC-I expression in VZV-infected HBVAFs stained with isotype for MHC-I (Iso), uninfected HBVAFs (UI) or VZV-infected HBVAFs. VZV gE negative bystander cells (Bys) or VZV gE positive cells (V+). (B) Representative flow cytometry plots for MHC-I mean fluorescence intensity (MFI) expression levels in HBVAFs. Iso = black histograms, UI = grey histograms, Bys = blue histograms and V+ = Red histograms. (C) Summary of MHC-I MFI expression levels in UI, Bys, V+ and Iso stained HBVAFs. Results are representative of three independent experiments. Bar graphs represent average MHC-I MFI levels ± SD. **P<0.01 and ****P<0.0001. # represents P<0.0001 for significant increases in MHC-I MFI levels in UI, Bys and V+ HBVAFs compared to Iso stained HBVAFs. Statistical significance was determined using ordinary one-way ANOVA with Tukey posttest. (TIF) [file ppat.1007650.s016.tif]

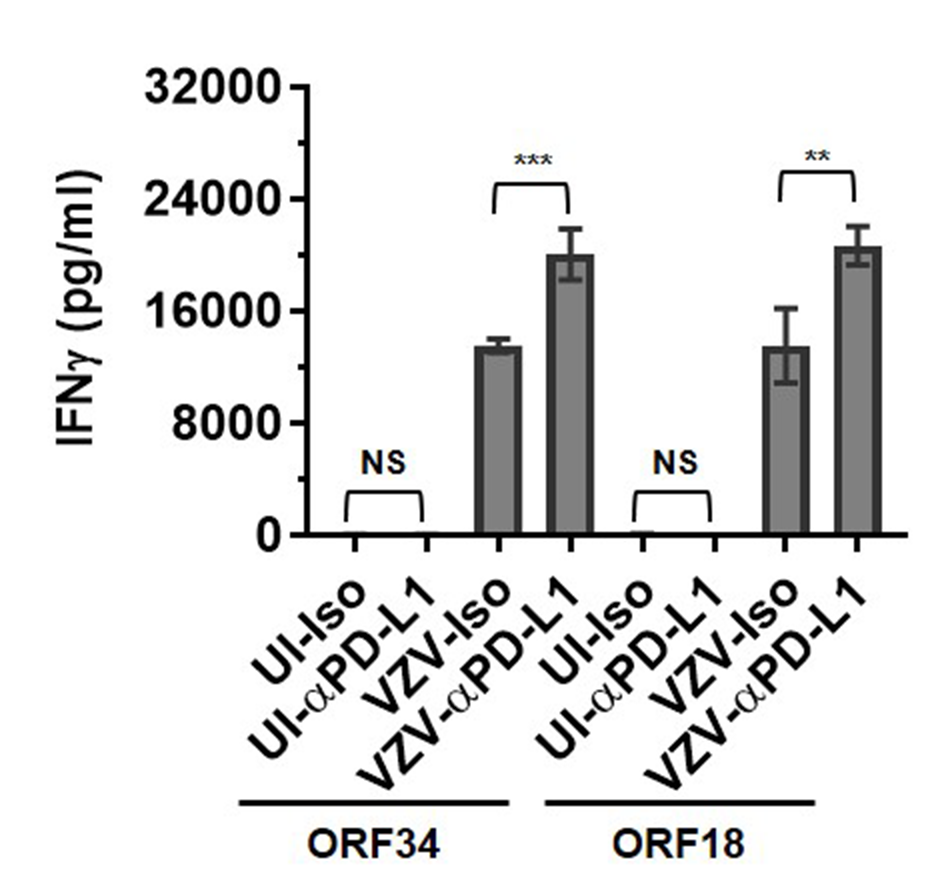

Supplement: S9 Fig — VZV ORF34- or ORF18-specific CD8+ T cells were co-cultured with uninfected- or VZV-infected HLA-A*0201+ HBVAFs for 24 h in the presence of PD-L1 blocking antibody (αPD-L1) or isotype controls. Then cell culture supernatants were harvested and analyzed for IFNγ levels using ELISA. Results are representative of 3 independent experiments with bar graphs representing average IFNγ levels (pg/ml) ± SD. **P<0.01, ***P<0.001. NS = not significant. Statistical significance was determined using RM one-way ANOVA with the Greenhouse-Geisser correction and Tukey posttest. (TIF) [file ppat.1007650.s017.tif]
